# PbrRALF2-elicited reactive oxygen species signaling is mediated by the PbrCrRLK1L13-PbrMPK18 module in pear pollen tubes

**DOI:** 10.1038/s41438-021-00684-y

**Published:** 2021-10-04

**Authors:** Xiaobing Kou, Jiangmei Sun, Peng Wang, Danqi Wang, Peng Cao, Jing Lin, Youhong Chang, Shaoling Zhang, Juyou Wu

**Affiliations:** 1 Center of Pear Engineering Technology Research, State Key Laboratory of Crop Genetics and Germplasm Enhancement, College of Horticulture, Nanjing Agricultural University, 210095 Nanjing, China; 2 Jiangsu Key Laboratory for Horticultural Crop Genetic Improvement, 210014 Nanjing, China

**Keywords:** Pollen tube, Plant signalling, Plant polarity, Plant molecular biology

## Abstract

Rapid alkalinization factors (RALFs) are cysteine-rich peptides that play important roles in a variety of biological processes, such as cell elongation and immune signaling. Recent studies in *Arabidopsis* have shown that RALFs regulate pollen tube growth via plasma membrane receptor-like kinases (RLKs). However, the downstream signal transduction mechanisms of RLKs in pollen tubes are unknown. Here, we identified PbrRALF2, a pear (*Pyrus bretschneideri*) pollen RALF peptide that inhibits pollen tube growth. We found that PbrRALF2 interacts with a malectin-like domain-containing RLK, PbrCrRLK1L13. The relative affinity between PbrRALF2 and PbrCrRLK1L13 was at the submicromolar level, which is consistent with the values of ligand–receptor kinase pairs and the physiological concentration for PbrRALF2-mediated inhibition of pollen tube growth. After binding to its extracellular domain, PbrRALF2 activated the phosphorylation of PbrCrRLK1L13 in a dose-dependent manner. We further showed that the MAP kinase PbrMPK18 is a downstream target of PbrCrRLK1L13 that mediates PbrRALF2-elicited reactive oxygen species (ROS) production. The excessive accumulation of ROS inhibits pollen tube growth. We show that MPK acts as a mediator for CrRLK1L to stimulate ROS production, which might represent a general mechanism by which RALF and CrRLK1L function in signaling pathways.

## Introduction

The pollen tube is a tubular structure used to transport sperm to the ovule for fertilization. This process requires crosstalk between pollen tubes and the pistil, which ensures that the pollen tube grows normally until reaching the ovule. The germination of a pollen grain and the subsequent rapid elongation of a pollen tube are regulated by multiple factors, such as extracellular peptides, hormones, and intracellular signals of calcium ions and reactive oxygen species. Among these factors, pollen-secreted peptides, which are multiple signaling molecules, play important roles in the development of pollen tubes, such as LAT52^1^, phytosulfokines^2^, and rapid alkalinization factors (RALFs)^3,4^. RALFs are cysteine-rich peptides (CRPs) of ~5 kDa^5^. Treatment of pollen with extracellular RALF inhibits pollen tube elongation, which is mediated through membrane receptors^4,6,7^.

BUPS1 and BUPS2 were recently identified as RALF receptors^4,6^, which are expressed in mature pollen grains and tubes. BUPS1 and BUPS2 belong to the rose periwinkle (*Catharanthus roseus*) receptor protein kinase (CrRLK1L) family, which is conserved in plants and has 17 members in *Arabidopsis*. CrRLK1Ls have been proposed to act as ‘sensor’ proteins. For example, FERONIA (FER) is a receptor for RALF1 and RALF23 in *Arabidopsis*, and it is involved in signaling for root elongation^8,9^, immunity^10^, and fertilization in female tissue^11,12^. In addition, the FER orthologs ANXUR1 (ANX1) and ANXUR1 (ANX2) are required to regulate pollen tube integrity^13–18^. Notably, the *bups1* and *bups1 bups2* loss-of-function mutants show precocious pollen tube rupture, similar to the phenotype of the *anx1 anx2* double mutant^6,14^. Despite this progress in understanding CrRLK1L family functions, the downstream signal transduction mechanisms of these receptor-like kinases in pollen tubes are poorly understood.

Mitogen-activated protein kinases (MPKs) are among the downstream targets of RALF signaling^5^, but the MPKs involved in the RALF response and how they are activated remain unknown. Some MPKs are known to be involved in transducing RLK signaling in other processes^19,20^; for example, MPK3 and MPK6 are downstream targets of (salt intolerance 1) SIT1 in rice and mediate salt-induced ethylene signaling^21^. Furthermore, a YODA-MKK4/MPK5-MPK3/MPK6 module regulates inflorescence architecture downstream of ERECTA-RLK^22^. ROS are also important messengers in RLK signaling^23^. ANX1 and ANX2 redundantly increase the activity of NADPH oxidases to generate ROS. ROS generation is indispensable for steady pollen tube elongation and helps to maintain the cell wall integrity of the pollen tube^24^. RALF17-based induction of ROS production is mediated by FER^10^. These results suggest that the suppression of pollen tube growth by RALF signaling could be determined by CrRLK1L-mediated ROS production.

In this study, we identified a pear pollen self-generated RALF peptide, PbrRALF2, and its receptor, PbrCrRLK1L13. We further found that after the direct interaction between ligand and receptor, PbrRALF2 promoted the phosphorylation of PbrCrRLK1L13, which subsequently recruited PbrMPK18 to produce ROS. The subsequent excessive accumulation of ROS inhibited pollen tube growth. The results indicate that a RALF-CrRLK1L-MPK-ROS pathway transduces extracellular signaling to the cytosol, thereby maintaining the moderate growth rate of the pollen tube in an autoregulatory manner.

## Results

### PbrRALF2 inhibits pear pollen tube growth

**Fig. 1:**
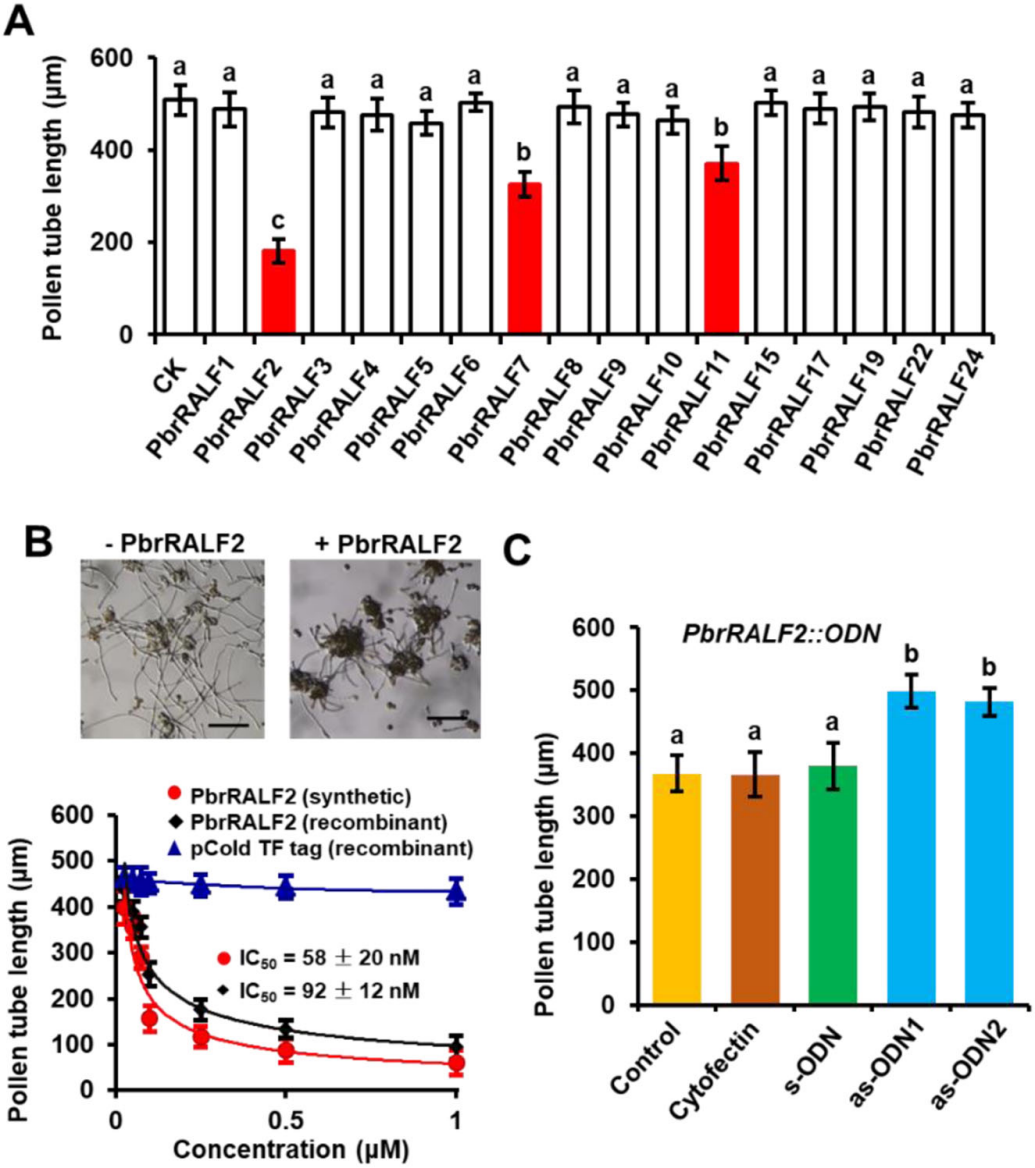
PbrRALFs inhibit pollen tube growth. **A** Pollen tubes from pear were treated with 1μM recombinant PbrRALFs, and pollen tube growth was measured. Selected PbrRALFs were expressed in pollen by RT-PCR (Supplementary Fig. S1A). Different letters indicate significant differences, as determined by one-way ANOVA (*P* *<* 0.05, *n* = 3). Three independent experiments were conducted. Data points are presented as the means ± s.e.m., with those significantly different from the control (CK) shown in red. **B** Recombinant or synthetic PbrRALF2 at different concentrations (0.025 μM, 0.05 μM, 0.075 μM, 0.1 μM, 0.25 μM, 0.5 μM, and 1 μM) was added to precultured pollen tubes. Images shown were acquired 10 h after treatment without or with purified recombinant PbrRALF2. Scale bars = 0.1 mm. Data points are presented as the means ± s.e.m. (IC_50_ = 58 ± 20 nM for synthetic PbrRALF2 and 92 ± 12 nM for recombinant PbrRALF2). **C** Knockdown (antisense oligodeoxynucleotide; as-ODN) of the expression of *PbrRALF2* promoted pollen tube growth (*P* *<* 0.05, *n* = 3), whereas s-ODN (sense ODN) and cytofection control had no function. UT indicates no treatment controls. Different letters indicate significant differences, as determined by one-way ANOVA (*P* *<* 0.05, *n* = 3). Three independent experiments were conducted

Using HMMER3 software with the RALF conserved domain PF05498 as a query, we identified 24 candidate *RALF* genes in the “Dangshansuli” pear genome and assigned them names based on the nomenclature and numbering conventions used for the *RALF* genes in *Arabidopsis* (Supplementary Table S1). Using reverse transcription PCR (RT-PCR), we found that 16 of the 24 *PbrRALF* genes were highly expressed in pollen (Supplementary Fig. S1A). We expressed and purified the 16 pollen-expressed PbrRALFs using an *Escherichia coli* system (Supplementary Fig. S1B). These purified recombinant proteins were used to treat pear pollen, and we observed that PbrRALF2, PbrRALF7, and PbrRALF11 significantly inhibited the growth of pollen tubes, with PbrRALF2 showing the largest effect (Fig. 1A). Based on these results, we selected PbrRALF2 for subsequent experiments.

*PbrRALF2* is expressed at a low level in multiple tissues, including the roots, stems, leaves, fruits, petals, and pistils, and is highly expressed in pollen (Supplementary Fig. S2A). To further explore the inhibition of pollen tube growth by PbrRALF2, we tested a synthetic PbrRALF2 peptide along with the purified recombinant PbrRALF2 described above for dose-dependent effects. The concentrations of PbrRALF2 required for 50% inhibition (IC_50_) of pollen tube growth were 58 ± 20 nM and 92 ± 12 nM for synthetic and *E. coli*-expressed PbrRALF2, respectively (Fig. 1B).

We next used an antisense oligonucleotide (as-ODN) approach to knock down gene expression, which is widely used in pollen tube genetic analysis^25–28^ to downregulate *PbrRALF2*. Significant promotion of pollen tube length was observed in PbrRALF2-as-ODN, where PbrRALF2 was knocked down, but not in the presence of the corresponding sense oligonucleotides (Fig. 1C). These results suggest that PbrRALF2 could inhibit pollen tube growth.

### PbrCrRLK1L13 is a receptor for PbrRALF2

To identify the components in PbrRALF2 signaling, we performed a yeast two-hybrid (Y2H) screen using PbrRALF2 as bait and a pear pollen cDNA library as prey. One of the candidates was Pbr001839.1 (Supplementary Table S2), which encodes PbrCrRLK1L13 of the pear CrRLK1L family^29^. CrRLK1Ls are membrane proteins that consist of an extracellular domain (ECD), a transmembrane domain (TM), and an intracellular kinase domain (IKD)^30^. Consistent with PbrCrRLK1L13 being a membrane protein, when we expressed PbrCrRLK1L13 in *Arabidopsis* protoplasts as a fusion with green fluorescence protein, it localized to the plasma membrane (Supplementary Fig. S3).

**Fig. 2:**
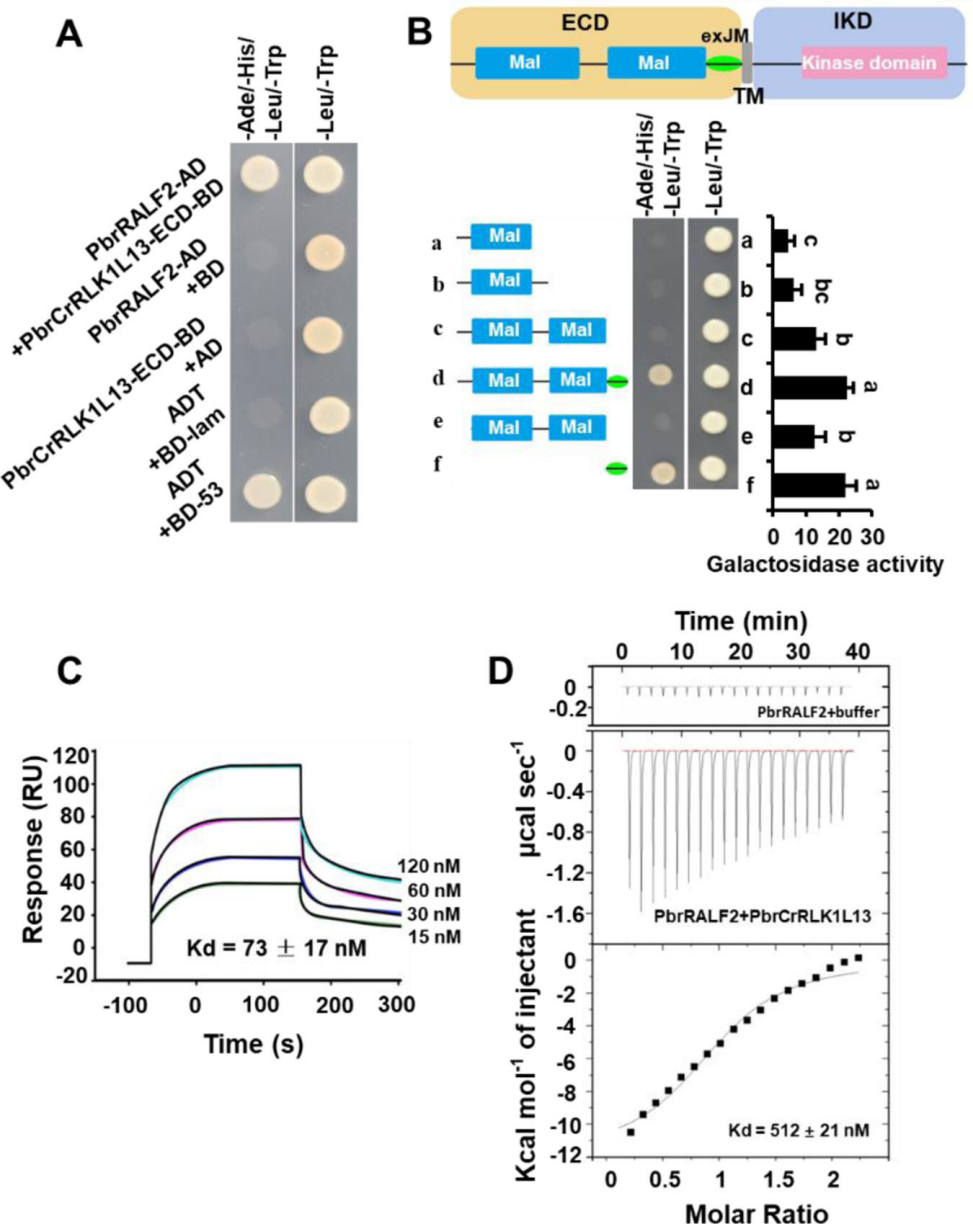
Binding of PbrRALF2 to PbrCrRLK1L13. **A** PbrRALF2 interacts with the extracellular domain of PbrCrRLK1L13 in yeast. PbrRALF2 was fused to the pGADT7 vector, and the extracellular domain of PbrCrRLK1L13 was fused to the pGBKT7 vector. Growth in the medium that lacked Trp, Leu, His, and Ade indicated protein–protein interactions. **B** Extracellular juxtamembrane (exJM) region of PbrCrRLK1L13 is required for the PbrRALF2 interaction. The predicted structure of PbrCrRLK1L13, including the extracellular domain (ECD), exJM, transmembrane domain (TM), and intracellular kinase domain (IKD). Dissection of the exJM region of PbrCrRLK1L13 is required for the interaction with PbrRALF2 in a yeast two-hybrid assay. The interaction strength was quantified through β-galactosidase activity. Different letters indicate significant differences, as determined by one-way ANOVA (*P* *<* 0.05, *n* = 3). **C** Binding of four concentrations of PbrRALF2 to PbrCrRLK1L13 in the surface plasmon resonance (SPR) assay. Different colored lines represent the titration of different concentrations of PbrRALF2 protein in the SPR assay. The light blue line indicates the 120 nM PbrRALF2 assay, the pink line indicates the 60 nM PbrRALF2 assay, the dark blue line indicates the 30 nM PbrRALF2 assay, and the green line indicates the 15 nM PbrRALF2 assay. The black lines represent the fitting line. The solid line is a fit with Michaelis–Menten kinetics, yielding an apparent dissociation constant of *K*d = 73 ± 17 nM. **D** Quantitative binding analysis using PbrRALF2 and the extracellular domain of PbrCrRLK1L13 using isothermal titration calorimetry (ITC). A representative thermogram was obtained from 200 μM PbrRALF2 titrations into 20 μM PbrCrRLK1L13. Nonlinear regression of the PbrRALF2 vs. PbrCrRLK1L13 dosage yielded an apparent dissociation constant of *K*d = 512 ± 21 nM

Although *PbrCrRLK1L13* from pear, *RUPO* from rice (Oryza sativa), and *BUPS1* (*At4g39110*) and *BUPS2* (*At2g21480*) from *Arabidopsis* belong to the same phylogenetic clade^29^, *RUPO*, *BUPS1*, and *BUPS2* have pollen-specific expression in rice^16^ and *Arabidopsis*^6^ while PbrCrRLK1L13 expression is observed in multiple tissues in pear (Supplementary Fig. S3A). To confirm the physical interaction between PbrRALF2 and PbrCrRLK1L13, we used two different approaches. A Y2H assay revealed that PbrRALF2 directly interacted with PbrCrRLK1L13 (Fig. 2A), and a deletion analysis delineated the interaction domain to the exJM of PbrCrRLK1L13 (Fig. 2B). No obvious interaction between PbrRALF2 and three other PbrCrRLK1L proteins, PbrCrRLK1L19 (homolog of THE1 in *Arabidopsis*), PbrCrRLK1L10 (homolog of HERK1 in *Arabidopsis*), and PbrCrRLK1L9 (homolog of HERK2 in *Arabidopsis*), was observed (Supplementary Fig. S4A). Moreover, both PbrRALF7 and PbrRALF11, the other two PbrRALFs that showed an inhibitory effect on pollen tube growth (Fig. 1A), also interacted with PbrCrRLK1L13 in Y2H assays (Supplementary Fig. S4B). Furthermore, we tested six PbrRALFs that showed no effects on pollen tube growth and found no interaction with PbrCrRLK1L13 (Supplementary Fig. S4C). Thus, these results support the direct interaction between PbrRALF2 and PbrCrRLK1L13.

We used surface plasmon resonance (SPR) and isothermal titration calorimetry (ITC) to determine the affinity of PbrRALF2 and PbrCrRLK1L13 for each other. The purified recombinant PbrRALF2 bound to PbrCrRLK1L13 with dissociation constant (*K*d) values of 73 ± 17 and 512 ± 21 nM, as revealed by SPR (Fig. 2C) and ITC (Fig. 2D), respectively. These values are consistent with those reported for other ligand–receptor kinase pairs^10,31^ and the IC_50_ for PbrRALF2-mediated inhibition of pollen tube growth (Fig. 1B). Thus, we conclude that PbrCrRLK1L13 could be a receptor for PbrRALF2.

### PbrRALF2 increases PbrCrRLK1L13 phosphorylation and ROS production

**Fig. 3:**
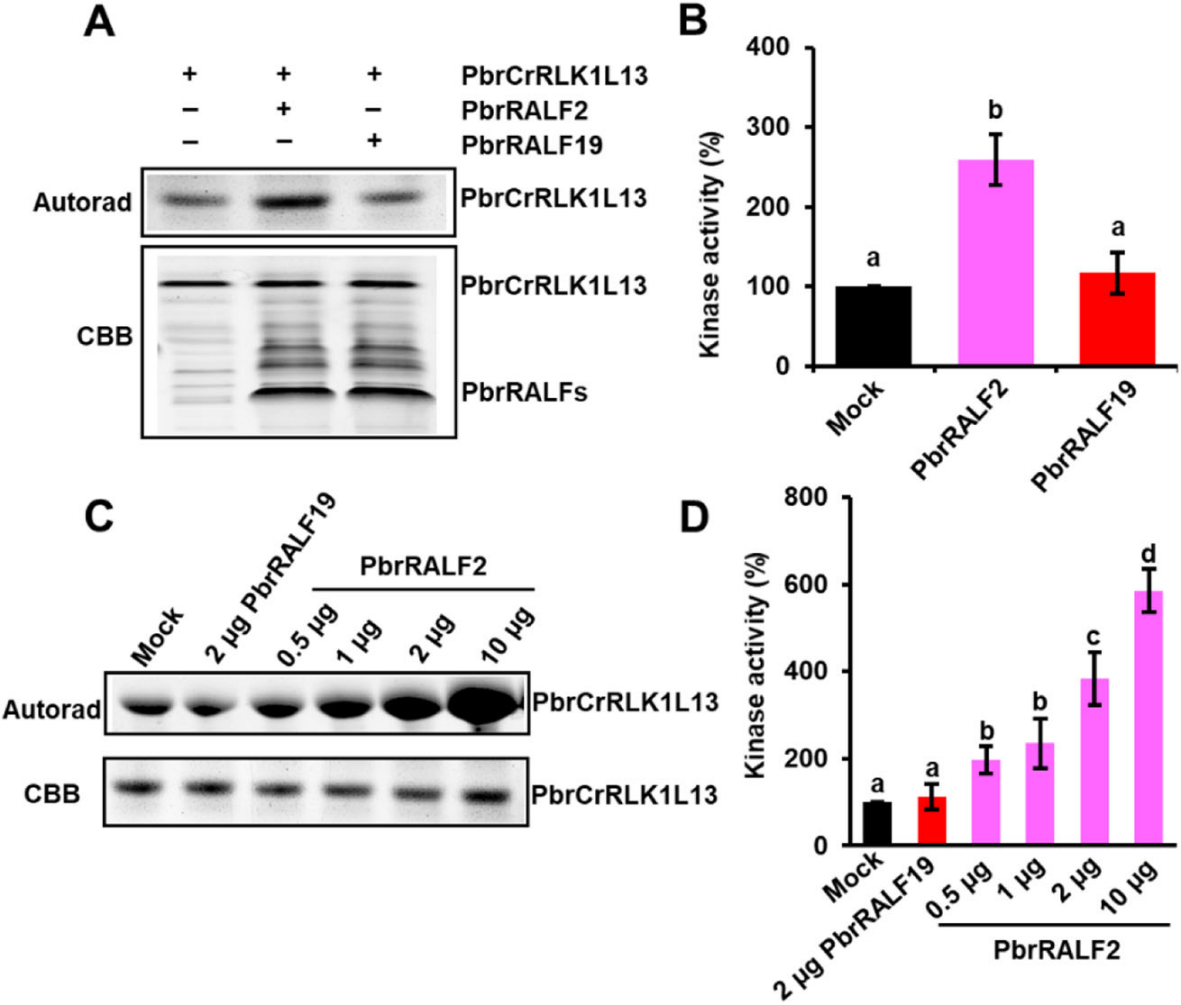
PbrRALF2 promotes PbrCrRLK1L13 phosphorylation. **A** In vitro kinase assay performed to test the kinase activity of PbrCrRLK1L13 treated with PbrRALF2 using ATP-γ-^32^P isotope labeling. *PbrRALF2* and *PbrRALF19* were cloned into the pCold-TF expression vector, and the full-length sequence of *PbrCrRLK1L13* was cloned into the pGEX-4T-1 expression vector. PbrCrRLK1L13 protein was treated with 1 μg PbrRALF2 or PbrRALF19 protein in kinase buffer along with 10 μCi ATP-γ-^32^P for 30 min at 30 °C. The denatured samples were subjected to SDS-PAGE and visualized using autoradiography with a phosphorimager (GE Healthcare, Chicago, IL, USA). **B** Quantitative analysis of radioactivity of the gel using the liquid scintillation counter. Samples were collected for the gels shown in (**A**). PbrRALF2 significantly promoted PbrCrRLK1L13 phosphorylation (*P* *<* 0.05, *n* = 3), whereas PbrRALF19 showed no significant effects. Data points are presented as the means ± s.e.m. Different letters indicate significant differences, as determined by one-way ANOVA. **C** Phosphorylation levels of PbrCrRLK1L13 increased by the PbrRALF2 treatments in a dose-dependent manner. PbrCrRLK1L13 was treated with 0.5, 1, 2, and 10 μg PbrRALF2 and 1 μg PbrRALF19 for 30 min. However, the phosphorylation levels of PbrCrRLK1L13 were not affected by PbrRALF19, even though the concentration of PbrRALF19 increased to 2 μg. **D** Quantitative analysis of the phosphorylation levels of PbrCrRLK1L13 treated with different concentrations of PbrRALF2. Samples were collected for the gels shown in (**C**). The histogram represents the relative intensity of the bands. The experiment was repeated three times. Different letters indicate significant differences, as determined by one-way ANOVA (*P* *<* 0.01, *n* = 3)

We further examined whether PbrCrRLK1L13 kinase activity is affected by PbrRALF2. PbrRALF2 could induce PbrCrRLK1L13 phosphorylation, suggesting that the binding of PbrRALF2 to PbrCrRLK1L13 is physiologically functional (Fig. 3A, B). The level of PbrCrRLK1L13 phosphorylation increased in a PbrRALF2 dose-dependent manner (Fig. 3C, D). However, the phosphorylation levels of PbrCrRLK1L13 were not affected by PbrRALF19, even when the concentration of PbrRALF19 was increased to 2 μg (Fig. 3C, D).

**Fig. 4:**
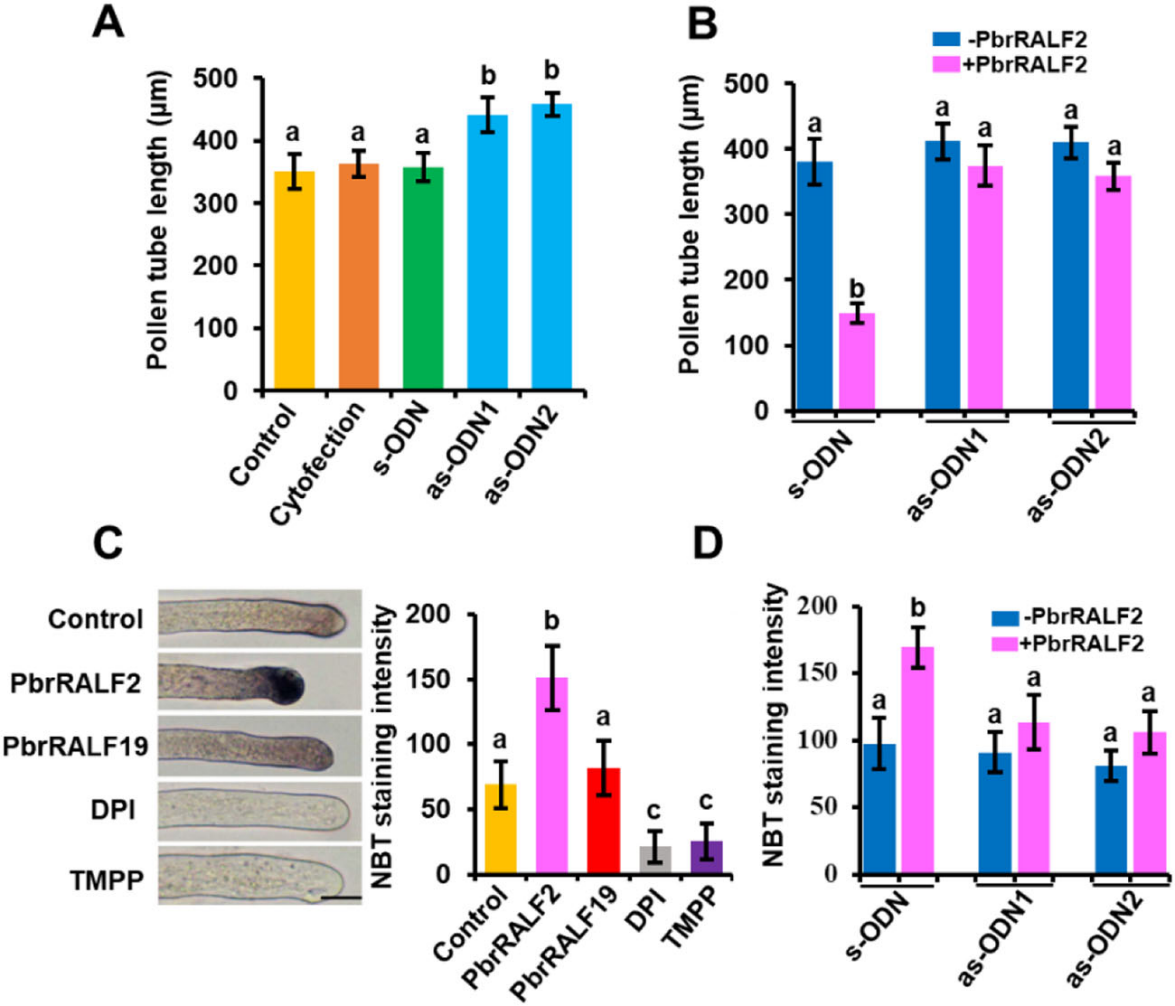
PbrRALF2-induced increase in reactive oxygen species and inhibition of pollen tube growth are mediated by PbrCrRLK1L13. **A** Knockdown of the expression of PbrCrRLK1L13 (as-ODN) promoted pollen tube growth (*P* *<* 0.05, *n* = 3), whereas the s-ODN and cytofection control did not. UT indicates no treatment controls. Thirty-five pollen tubes were measured in each of three independent experiments. Different letters indicate significant differences, as determined by one-way ANOVA. **B** Inhibition of pollen tube growth was not sensitive to PbrRALF2 in the PbrCrRLK1L13 knockdown group compared with the significant inhibition of pollen tube growth in the s-ODN control group (*P* *<* 0.05, *n* = 4). Three independent experiments were conducted. Different letters indicate significant differences, as determined by two-way ANOVA. **C** PbrRALF2 induces reactive oxygen species (ROS) production in pollen tubes. Representative images of pollen tubes incubated with nitroblue tetrazolium (NBT) under different treatments for 30 min (left panel). Scale bar = 40 μm. Experiments were repeated at least three times with similar results. PbrRALF2 significantly increased the mean pixel intensity of the pollen tube tip (*P* *<* 0.05, *n* = 3; right panel), which indicated an increase in ROS, but PbrRALF19 did not. Different letters indicate significant differences, as determined by one-way ANOVA. **D** PbrRALF2 did not increase ROS in the as-ODN-treated PbrCrRLK1L13 pollen tube tips. Three independent experiments were conducted. Different letters indicate significant differences, as determined by two-way ANOVA

We observed a significant promotion of pollen tube length in the presence of as-ODN targeted to the *PbrCrRLK1L13* sequence but not in the presence of s-ODN (Fig. 4A). Moreover, the growth of the *PbrCrRLK1L13*-knockdown pollen tube was less sensitive to PbrRALF2 (Fig. 4B). Similar to RALF17, which induces the production of ROS in *Arabidopsis*^10^, ROS production was significantly increased upon PbrRALF2 treatment in pear pollen (Fig. 4C and Supplementary Fig. S7A). Consistent with the lack of phosphorylation of PbrCrRLK1L13 in the presence of PbrRALF19, ROS production was also insensitive to PbrRALF19. Notably, when the expression of *PbrCrRLK1L13* was knocked down, ROS production became insensitive to PbrRALF2 (Fig. 4D and Supplementary Fig. S7B). Together, these results suggest that PbrCrRLK1L13 mediates the PbrRALF2-elicited increase in ROS production and pollen tube growth inhibition in pear.

### PbrMPK18 is one of the downstream targets of PbrCrRLK1L13

**Fig. 5:**
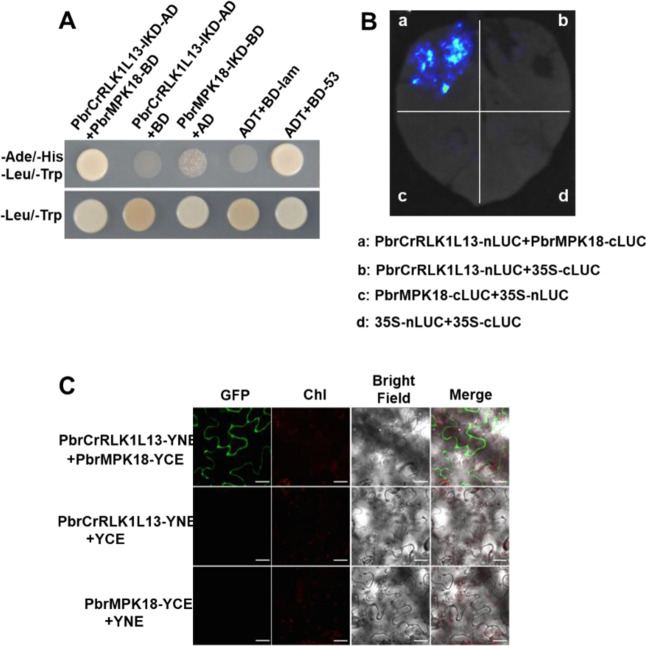
PbrMPK18 interacts with PbrCrRLK1L13. **A** PbrMPK18 interacts with the intracellular domain of PbrCrRLK1L13 in yeast two-hybrid assays. The intracellular domain of PbrCrRLK1L13 fused to the pGADT7 vector and that of PbrMPK18 fused to the pGBKT7 vector. Growth in the medium that lacked Trp, Leu, His, and Ade indicated protein–protein interactions. **B** PbrMPK18 and PbrCrRLK1L13 interact in the plasma membrane of *N. benthamiana* leaf epidermal cells in the LCI assay. Fluorescence on the leaf surface indicated protein–protein interactions. **C** PbrMPK18 and PbrCrRLK1L13 interact in the plasma membrane of *N. benthamiana* leaf epidermal cells in the BiFC assay. The GFP signal indicates the interaction between PbrMPK18 and PbrCrRLK1L13. Chl represents chloroplast autofluorescence. Merge represents the merged image of GFP, Chl, and bright field. Scale bars = 5 μm

**Fig. 6:**
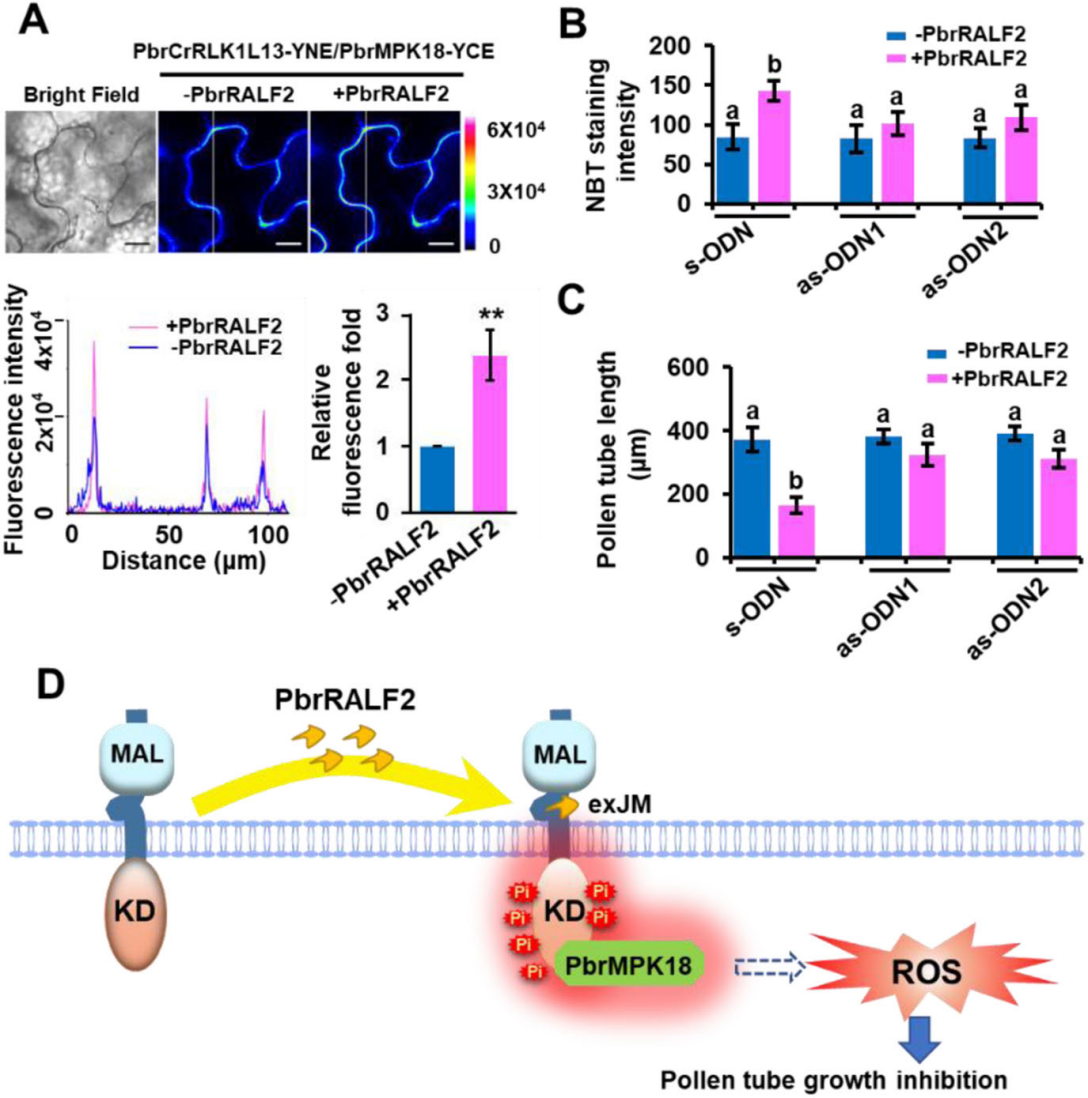
PbrRALF2 enhances the PbrCrRLK1L13-PbrMPK18 interaction, and PbrMPK18 is required for PbrRALF2 signaling. **A** Interaction of PbrCrRLK1L13 with PbrMPK18 analyzed by BiFC in *N. benthamiana* epidermal cells. The interaction levels of PbrCrRLK1L13 and PbrMPK18 were significantly enhanced by 1 μM PbrRALF2. (** represents *P* *<* 0.01; Student’s *t* test). Data points are presented as the means ± s.e.m. Scale bar = 20 μm. **B** PbrMPK18 is required for PbrRALF2-induced ROS accumulation. PbrMPK18 was knocked down by as-ODN1 and as-ODN2, with s-ODN as a negative control. PbrRALF2 (1 μM) was used to treat the pollen tubes. ROS accumulation was tested by NBT staining. Thirty-five pollen tubes were measured in each of three independent experiments. Different letters indicate significant differences, as determined by two-way ANOVA. **C** Inhibition of pollen tube growth was not sensitive to PbrRALF2 when PbrMPK18 was knocked down by as-ODN in pollen tubes. Thirty-five pollen tubes were measured in each of three independent experiments. Different letters indicate significant differences, as determined by two-way ANOVA. **D** Proposed model for PbrRALF2 perception in pollen tubes of pear. After PbrRALF2 binds to the exJM region of PbrCrRLK1L13, phosphorylation of PbrCrRLK1L13 is enhanced. Under the mediation of PbrMPK18, phosphorylated PbrCrRLK1L13 increases ROS production in pollen tubes. Excessive ROS production eventually inhibits the growth of pollen tubes

To identify intracellular downstream signaling molecules of PbrCrRLK1L13 in pollen tubes, we used the PbrCrRLK1L13 kinase domain as bait to screen a cDNA library of pear pollen tubes by Y2H. PbrMPK18 was identified as an interactor of PbrCrRLK1L13 (Supplementary Table S3). The Y2H assay (Fig. 5A), luciferase complementation imaging (LCI) (Fig. 5B), and bimolecular fluorescence complementation (BiFC) assays (Fig. 5C) revealed that PbrMPK18 interacts directly with PbrCrRLK1L13. The interaction level of PbrCrRLK1L13 and PbrMPK18 was significantly enhanced in the presence of PbrRALF2 (Fig. 6A). Conversely, when *PbrMPK18* was knocked down using the antisense oligonucleotide approach, both ROS production (Fig. 6B and Supplementary Fig. S7C) and pollen tube growth (Fig. 6C) became insensitive to PbrRALF2. Thus, PbrMPK18 acts as a downstream component of PbrCrRLK1L13 to mediate ROS production and pollen tube growth inhibition in PbrRALF2 signaling (Fig. 6D).

## Discussion

### PbrRALF2 acts as an inhibitor to control the balance of pollen tube growth

RALFs have long been identified as ubiquitous peptides in plants. Inhibitory effects of RALFs on roots^5,32^ and pollen tube growth have also been observed^3,4,6^. In this study, we identified a signaling pathway for PbrRALF2 that inhibits the growth of pear pollen tubes. Our results support a model in which PbrRALF2 is secreted into the apoplast during pollen tube elongation and then binds to PbrCrRLK1L13 and triggers its intracellular phosphorylation. This process leads to the inhibition of pollen tube elongation and thus controls the steady-state growth of the pollen tube.

Several lines of evidence suggest an inhibitory effect of PbrRALF2 on pollen tube growth. Both synthetic and purified *E. coli*-expressed PbrRALF2 induced dose-dependent inhibition of pollen tube elongation (Fig. 1B). When *PbrRALF2* was knocked down, the pollen tubes grew faster (Fig. 1C). The finding that PbrRALF2 inhibits pollen tube growth was initially puzzling because this peptide from pollen was produced by itself. However, it is possible that this growth inhibition is a form of autoregulation that allows the pollen tube to grow normally. Other pollen self-generated peptides are known to contribute to this type of regulation^2^, and accurate control of the rate of pollen tube growth is essential for successful fertilization.

### RALF signal was transduced into the cytosol via PbrCrRLK1L13

Many peptides have been identified as apoplast signals, which exert their function using membrane receptors. RLKs are one such type of receptor in plants involved in the perception of extracellular signals and cytoplasmic interactor transduction^33^. For example, CLAVATA1, the receptor of CLV3, controls the size of the organizing center in the shoot apical meristem and regulates the expansion of plant roots^34,35^; and PSKR, as the receptor of PSK, regulates cell expansion during plant development^36^. In addition, pollen-specific RLK proteins regulate pollen tube growth. The receptor kinase LePRK2 transduces LAT52 information to control pollen hydration and germination^37^. A pollen-specific receptor kinase in *Arabidopsis* is essential for pollen tube reception of the signal from the AtLURE1 peptide^38,39^. FER is a receptor for AtRALF1 to inhibit cell elongation in roots^8,9^ and AtRALF23 to inhibit plant immunity^10,40^. Here, we provide evidence that PbrCrRLK1L13 is a receptor for PbrRALF2 in pear pollen. This result represents another ligand–receptor pair involving a RALF and a CrRLK1L protein and highlights the diverse functions of this complex in regulating plant growth and cell–cell communication. PbrRALF2 can bind to PbrCrRLK1L13 and has submicromolar affinity (*K*d) for PbrCrRLK1L13 (Fig. 2), thus meeting the criterion of ligand–receptor pairs^13,41^, and this affinity is consistent with the physiological concentration required for its inhibition of pollen tube growth (Fig. 1B).

Notably, *PbrCrRLK1L13* belongs to the same clade as *BUPS1* and *BUPS2* of *Arabidopsis* and *RUPO* of rice^15,16,29^. Based on our evidence and previous reports, we know that PbrCrRLK1L13, BUPS1, and BUPS2 are receptors for RALF peptides^6^ (Fig. 2). RUPO is known to facilitate male transmission by controlling the potassium concentration in pollen tube^16^, but further investigation is required to determine whether RUPO is also a receptor of RALF in the pollen tube of rice.

PbrCrRLK1L13 interacts with its PbrRALF2 ligand via exJM at the plasma membrane (Fig. 2B), similar to the region where LORELEI and LLG1 physically interact with FER^42^, thus implying a potentially conserved role for the exJM region of CrRLK1Ls. The phosphorylation of PbrCrRLK1L13 can be directly promoted by PbrRALF2 (Fig. 3), and knockdown of *PbrCrRLK1L13* renders the pollen tube less sensitive to PbrRALF2 (Fig. 4B), indicating that the interaction of PbrRALF2 with PbrCrRLK1L13 is functionally relevant. Such phosphorylation by peptides has similarly been observed for multiple receptor-like kinase proteins. In *Arabidopsis*, RALF1 triggers FER^8^ and BAK1^41^ phosphorylation to suppress root elongation. In the self-incompatibility response of *Brassica*, the pollen *S*-determinant *S* locus cysteine-rich protein binds to its corresponding pistil *S*-determinant *S* locus receptor kinase (SRK), resulting in the phosphorylation of SRK and initiation of a signal transduction cascade that ultimately results in pollen death^43–46^. Considering these results together, it seems possible that phosphorylation of the cytoplasmic kinase domains of membrane RLKs represents a general method of conveying extracellular peptide messages to cytosolic proteins.

### Enhanced PbrCrRLK1L13-induced ROS production is mediated by PbrMPK18

MAP kinases can be activated by RALFs^5^, but it is not clear how RALFs transmit signals to MAPKs and which components encode RALF-responsive MAPKs. In addition to MKKs, MAPKs are also regulated by other kinases^47–49^. Here, interaction studies revealed the binding of PbrCrRLK1L13 to PbrMPK18 (Fig. 5), indicating that PbrMPK18 should be considered a downstream target of PbrCrRLK1L13. Importantly, the interaction of PbrCrRLK1L13 with PbrMPK18 was enhanced by the PbrRALF2 treatment (Fig. 6A). When *PbrMPK18* was knocked down, pollen tube growth was insensitive to PbrRALF2 (Fig. 6C), thus providing more direct evidence that PbrMPK18 plays a key role in PbrRALF2 signaling. PbrMPK18 is closely related to *Arabidopsis* MPK6; thus, further work will be interesting to investigate whether MPK6 is involved in RALF signaling in *Arabidopsis*.

A central element of RLK signaling is the ROS burst^23,50^. In FER-mediated signaling, RALF17 causes an increase in ROS production; however, RALF peptides have a predicted S1P cleavage site that leads to an inhibition of ROS production, and the specific mechanisms underlying the differences among RALFs remain unclear^10^. ROS production is essential for cell signaling and regulation, although too much ROS can be harmful to cell homeostasis. At low levels, ROS can alter the intracellular redox state, activate redox-sensitive proteins, and modify redox-sensitive domains of proteins by inhibiting or boosting their enzymatic activity^51,52^, while at high levels, ROS damage DNA, proteins, phospholipids, and other macromolecules, thereby impairing physiological function^53^. Two classes of signaling components have been suggested as the main mediators of RLK stimulation of extracellular ROS production^23^. One is receptor-like cytoplasmic protein kinases, such as BIK, which mediate FLS2 and BIR1 signaling to produce ROS. The other includes small guanine nucleotide-binding proteins, such as GEF1, which mediate FER and AtPRK2 signaling to produce ROS. We observed here that activation of PbrCrRLK1L13 led to the production of ROS in a manner mediated by PbrMPK18 (Fig. 6B). These findings point to another mechanism for RLK-induced ROS production. However, the connection between MAPKs and ROS signaling remains elusive, and future work is necessary to unravel the underlying mechanisms.

Based on our results, we propose a model for a signaling event in which peptide–receptor interactions lead to the suppression of pollen tube growth (Fig. 6D). During pollen tube elongation, PbrRALF2 is predicted to bind to the exJM of receptor PbrCrRLK1L13, thereby promoting its intracellular phosphorylation. In a process mediated by PbrMPK18, the PbrRALF2–PbrCrRLK1L13 complex enhances ROS production, thereby leading to the suppression of pollen tube growth. Our results thus elucidate events during pollen tube growth and suggest that CrRLK1Ls generally serve as receptors for RALFs in plants.

## Materials and methods

### Plant materials

Root, stem, leaf, petal, fruit, pollen, and pistil tissues from *Pyrus bretschneideri* Rehd. cv. Danshansuli pear trees were collected from the Fruit Experimental Yard of Nanjing Agricultural University. Pollen samples were preserved by air-drying at ambient temperature (25 °C) for 12 h and subsequently stored at −20 °C with silica gel. Root, stem, leaf, petal, fruit, and pistil samples were frozen in liquid nitrogen and stored at −80 °C for RNA extraction.

Mesophyll protoplasts for protein subcellular localization analysis were extracted from *Arabidopsis thaliana* (Col-0) leaves grown under short-day (8 h light and 16 h dark at 22 °C) conditions^54^. *Nicotiana benthamiana* seedlings were grown in the greenhouse with cycles of 16 h light and 8 h dark at 25 °C.

### Identification of *RALF* and *CrRLK1L* genes in pear

To identify members of the *RALF* gene family in pear, the RALF domain sequence (PF05498) was used as a query for searching against the pear genome database (http://peargenome.njau.edu.cn) with the HMMER3 software package. We identified 24 *RALFs* in pear. All *RALF* genes with nonredundant hits and expected *E* values <0.01 were collected. The predicted RALF proteins were confirmed using Pfam (http://pfam.sanger.ac.uk/search) and SMART (http://smart.embl-heidelberg.de/) tools.

### Expression and purification of the His–PbrRALF fusion protein

The mature coding sequences for *PbrRALF* genes were amplified using PCR with BamHI and XbaI sites at the 5′- and 3′-ends; the primers for *PbrRALFs* are listed in Supplementary Table S4. The amplified PCR products were digested by BamHI and XbaI and ligated into the pCold-TF DNA vector (Takara Bio, Dalian, China). The recombinant plasmids were individually transferred into *E. coli* BL21 (DE3) cells. Expression of the fusion protein (His–PbrRALFs) was induced by isopropylthio-β-d-1-galactopyranoside (IPTG, 0.5 mM) after the culture reached an OD_600_ of 0.6 at 37 °C. The cultivation was continued at 15 °C for 24 h with continuous shaking (220 rpm), and the cells were subsequently collected by centrifugation for protein purification. The bacterial pellet from the 600 mL culture was resuspended in 30 mL lysis buffer (50 mM NaH_2_PO_4_, 300 mM NaCl, 10 mM imidazole, pH 7.9) and subsequently disrupted using an ultrasonic cell cracker (Model 705, Fisher Scientific, USA). The extract was centrifuged at 10,000×*g* for 15 min at 4 °C, and the supernatant was recovered. The supernatant containing soluble proteins was added to a column containing 4 mL Ni-NTA His Bind Resin (EMD Millipore, MA, USA) and washed with 20 mL binding buffer (20 mM Tris-HCl, 500 mM NaCl, 10 mM imidazole, pH 7.9), 5 mL wash buffer (20 mM Tris-HCl, 500 mM NaCl, 100 mM imidazole, pH 7.9), and 3 mL elution buffer (20 mM Tris-HCl, 500 mM NaCl, 600 mM imidazole, pH 7.9). The purified proteins were stored at −80 °C. Protein quality was assessed by SDS/PAGE.

### PbrRALF treatment assay

First, the preservation solution of PbrRALFs was dialyzed (dialysis membrane, molecular cutoff 6,000–8,000) against 2 L of pollen culture medium ((w/v), 0.03% Ca(NO_3_)_2_.4H_2_O, 10% sucrose, 0.01% H_3_BO_3_, and 30 mM MES, pH 6.3) at 4 °C for 16 h. Subsequently, pear pollen samples were precultured in the basal medium at 25 °C in darkness for 40 min. Subsequently, a portion of the precultured pollen tubes was treated with PbrRALFs for 2 h, and equivalent His-TF expressed from empty pCold-TF DNA vectors were used as the controls. The pollen tubes were photographed using an Olympus IX73 microscope (Olympus Optical, Japan, https://www.olympus-lifescience.com). The length of the pollen tubes was measured with ImageJ software (https://imagej.nih.gov/ij).

### Quantitative RT-PCR

The MIQE guidelines were followed for quantitative RT-PCR (qRT–PCR) experiments. The Plant Total RNA Isolation Kit (FOREGENE, Chengdu, China) was used to extract total RNA, and DNase I was used to eliminate genomic DNA contaminants. A RevertAid First Strand cDNA Synthesis Kit was used to reverse transcription purified total RNA (3 μg) (Thermo Scientific, USA). Then, using SYBR Green Master Mix on a LightCycler^®^ 480 II (Roche, Switzerland), quantitative PCR (qPCR) was performed on 20-μl samples according to the manufacturer’s instructions. The thermal cycle was performed at 95 °C for 5 min, followed by 45 cycles of 15 s at 95 °C and 15 s at 60 °C. The pear TUBULIN gene was used as an internal control, and relative expression levels were evaluated using the 2CT2 − △△CT method, as previously described^55^. The primers for RT-PCR and qRT–PCR of PbrRALFs, PbrCrRLK1L13, PbrMPK18, and PbrTUB in pear are presented in Supplementary Tables S5 and S6, respectively.

### ROS detection assays in pollen tubes

To detect ROS production in the tip of the pollen tube after PbrRALF2 treatment, we used PbrRALF2 to treat pear pollen for 2 h at 25 °C. PbrRALF19 was used as a negative control. Pollen tubes were stained with nitroblue tetrazolium (NBT; 1 mg mL^−1^) for 5 min^56^. The stained samples were photographed using an Olympus IX73 microscope. ImageJ software was used to calculate the pixel intensity of formazan precipitation in the pollen tube tips. The pixel intensity values were calculated as previously described^57^. More than 35 pollen tubes were measured in each treatment, with three biological replicates. In addition, we further used 2′,7′-dichlorodihydrofluorescein (CM-H2DCFDA, final concentration 5 μM) to detect ROS production. The pollen tubes were then imaged by confocal microscopy on a Zeiss 880 LSCM microscope.

### Subcellular localization analysis

The full-length coding sequence of pear *PbrCrRLK1L13* was amplified using PCR from pear pollen cDNA with Phusion High-Fidelity DNA Polymerase (Thermo Scientific, USA). The following primers were synthesized with restriction sites: PbrCrRLK1L13 forward, 5ʹ-GCTCTAGAATGGCTCTCCTCCTGGTCCT-3ʹ, and PbrCrRLK1L13 reverse, 5ʹ-GAAGATCTCCTACCATTTAGATTGGAAAATTG-3ʹ. The PCR products for PbrCrRLK1L13 were subsequently digested using XbaI and BglII. The digested PCR products were fused in frame with the sequence encoding GFP into XbaI and BamHI sites of the *p35S:CDS-GFP* vector^58^ to produce the plasmid *p35S:PbrCrRLK1L13-GFP*, which was then transfected into *Agrobacterium* (GV3101). *Agrobacterium*-mediated infiltration in *Nicotiana benthamiana* leaves was performed with a needleless syringe as reported^59^. The images were acquired using a Zeiss LSM 780 (Germany, https://www.zeiss.com/microscopy) confocal laser-scanning microscope after 3 days of tobacco cultivation. All transient expression assays were repeated at least three times.

### Yeast two-hybrid assay

*PbrRALF2* was cloned into a pGBKT7 vector and used as bait in the AH109 strain to screen the pear pollen cDNA library. Yeast two-hybrid analyses were conducted as previously described^60^, and PbrCrRLK1L13 was identified as a candidate protein. To verify the interaction between PbrRALF2 and PbrCrRLK1L13, *PbrRALF2* was cloned into the pGADT7 vector, and the sequence encoding the PbrCrRLK1L13 extracellular domain (aa 1–417) was cloned into the pGBKT7 vector. The constructed vectors were cotransformed into yeast, selected on SD/–Leu–Trp and verified on SD/–Leu–Trp–His–Ade. Then, the PbrCrRLK1L13 kinase domain (aa 441–770) was used as bait to screen the pear pollen cDNA library using the AH109 system. The method of screening and validation in yeast was similar to the interaction between PbrRALF2 and PbrCrRLK1L13. The primers used for the yeast two-hybrid assay are listed in Supplementary Table S7.

### Bimolecular fluorescence complementation

For the BiFC^61^ assay, the coding sequence of *PbrMPK18* was cloned into pSPYCE-35S, and the full-length sequence of *PbrCrRLK1L13* was cloned into pSPYNE-35S. *Agrobacterium* carrying the plasmid were coinfiltrated in *N. benthamiana* leaves. Confocal microscopy (Zeiss LSM 780, Germany, https://www.zeiss.com/microscopy) images were captured 3 days after infiltration. The primers used for gene cloning and vector construction are listed in Supplementary Table S8.

### Luciferase complementation imaging assay

A luciferase complementation imaging (LCI) assay was conducted. The full-length coding sequence of PbrCrRLK1L13 was cloned into the vector nLUC, and the full-length coding sequence of PbrMPK18 was cloned into the vector cLUC. Paired constructs of PbrMPK18-cLUC and PbrCrRLK1L13-nLUC were transiently coexpressed in the leaves of *N. benthamiana* through *Agrobacterium*-mediated coinfiltration. The primers used for this assay are listed in Supplementary Table S9.

### Expression and purification of GST-PbrCrRLK1L13-ECD

The region encoding the extracellular domain of PbrCrRLK1L13 was amplified by PCR and cloned into the pGEX-4T-1 vector. The corresponding primers with BamHI and XhoI sites were as follows: forward, 5ʹ-CGGGATCCATGGCGTCCTCCCCACC-3ʹ, and reverse, 5ʹ-CCGCTCGAGCTTGTGAGGCCCGCCG-3ʹ. The expression vectors were transformed into *E. coli* BL21 (DE3) and subcultured in 600 mL of Luria–Bertani (LB) medium until the OD_600_ reached 0.6. Then, the expression of GST (from the empty vector) or GST-PbrCrRLK1L13-ED was induced by IPTG (0.5 mM), and the cells were further cultivated for 8 h at 26 °C. One milliliter of binding buffer (25 mM Tris•HCl, pH 7.5, 100 mM NaCl, and 1 mM DTT) was used to prewash GST agarose resin (Thermo Fisher Scientific, USA) three times.

### In vitro phosphorylation assays

The full-length sequence of *PbrCrRLK1L13* was cloned into the pGEX-4T-1 expression vector to produce GST-tagged recombinant protein in *E. coli*. Primers for *PbrCrRLK1L13* were as follows: forward, 5ʹ-GAAGATCTATGGCTCTCCTCCTGGTCC-3ʹ, and reverse, 5ʹ-CCGCTCGAGTTACCTACCATTTAGATTGGAAAAT-3ʹ. The membrane proteins were expressed using MembraneMax™ Protein Expression Kits (Thermo Fisher Scientific, USA). Ni-NTA His Bind resin (EMD Millipore, MA, USA) was used to purify the protein, and then SDS/PAGE was performed to determine the protein purity. The reaction buffer was [25 mM Tris•HCl (pH 7.5), 1 mM MnSO_4_, 0.5 mM CaCl2, 2 mM DTT, 10 µM ATP, and 10 µCi of [y-32] ATP (3000 Ci mmol-1)]. Kinase activity assays were conducted for 30 min at 30 °C. SDS-PAGE was used to analyze the denatured samples, which were then stained with Coomassie brilliant blue and autoradiographed with a phosphorimager (GE Healthcare, USA).

### Surface plasmon resonance assay

Surface plasmon resonance (SPR) experiments were conducted at 25 °C using a Biacore T200 (GE Healthcare, USA, https://www.biacore.com). On a CM5 sensor chip, PbrCrRLK1L13 immobilization was accomplished using the standard amine coupling technique with a running buffer of 10 mM HEPES, 150 mM NaCl, and 3 mM EDTA at pH 7.4. With a contact time of 60–100 s, a flow rate of 100 L/min, and a dissociation time of 200–300 s, PbrRALF2 was injected onto the surface. By subtracting the signals on the reference surface from those on the target protein surface and the signals of the blank sample (no mimetics) from those of the mimetic-containing samples, the sensorgrams were double-corrected for nonspecific binding to the surface. With Biacore T200 Evaluation Software (version 2.0), the corrected signal was fitted to a steady-state 1:1 interaction model and the binding affinity (Kd) was obtained. GraphPad Prism was used to assess differences in Kd values for each test using one-way ANOVA and multiple Tukey’s comparison tests (version 6.05).

### Isothermal titration calorimetry assay

A MicroCal ITC200 instrument (Malvern Instruments, England, https://www.malvern.com) was used to acquire isothermal titration calorimetry (ITC) data at 25 °C. Using a PD-10 column, a 20 μM PbrCrRLK1L13 solution was prepared in 50 mM phosphate, pH 7.4, and 150 mM NaCl (GE Healthcare, USA). PbrRALF2 solutions were produced as 200 μM stocks in the same buffer. The sample cell was filled with 400 μL of PbrCrRLK1L13 solution, and the syringe was filled with 40 μL of PbrRALF2 solution in each ITC experiment. The PbrCrRLK1L13 solution was stirred at 1000 rpm. The titration began with a 0.2 μL injection, followed by a series of 29 periodic injections of 1 μL each. The data were analyzed using the evaluation software Origin 7 (MicroCal Inc., USA). Thermodynamic parameters were calculated using the “one set of sites” model and a nonlinear least-squares fit to the data.

### Gene knockdowns by antisense oligonucleotides (as-ODN)

Twenty-mer phosphorothioate oligonucleotides against *PbrRALF2*, *PbrCrRLK1L13*, *PbrMPK18*, and their sense controls (s-ODN) were synthesized with three 5′- and 3′-phosphorothioate-modified bases, and a scrambled mismatch sequence was used as a control. For the in vitro experiments, pear pollen grains were precultured at 25 °C in darkness for 45 min in basal medium and pretreated with as-ODN and s-ODN (30 μM final concentration) with cytofectin (15 μg/ml) for 15 min, with as-ODN and s-ODN injected in the pollen medium. After 2 h of cultivation, the samples were photographed using an Olympus IX73 microscope. More than 35 pollen tubes were measured in each treatment, and the experiments were repeated at least three times. The ODN sequences are shown in Supplementary Table S10. For each gene, two as-ODNs were designed for the test. Two independent as-ODN1 and as-ODN2 with higher gene knockdown efficiency were chosen for further experiments (Supplementary Figs. S2B, S3B, and S6).

### Statistical analysis

All experimental data represent at least three independent experiments and are presented as the mean ± standard error (SE). GraphPad Prism (version 6.05) was used to analyze the data, and significant differences were compared using the Student’s *t* test for two groups of samples and ANOVA for multiple samples. Tukey’s honestly significant difference test was used for multiple comparisons. ANOVA tables are provided in Supplementary Data S1.

## Supplementary information

Supplementary information

## Data Availability

The authors confirm that the data in this study are available within the article and its supplementary materials. Original data can be requested from the corresponding authors.
