# Supplementary material for: PbrRALF2-elicited reactive oxygen species signaling is mediated by the PbrCrRLK1L13-PbrMPK18 module in pear pollen tubes
Source: Hortic Res. 2021 Oct 4;8:222. doi: 10.1038/s41438-021-00684-y (PMC8490453; doi:10.1038/s41438-021-00684-y)
Supplement: Supplementary file 1 — Supplementary information [file 41438_2021_684_MOESM1_ESM.docx]

**Supporting Information**

**
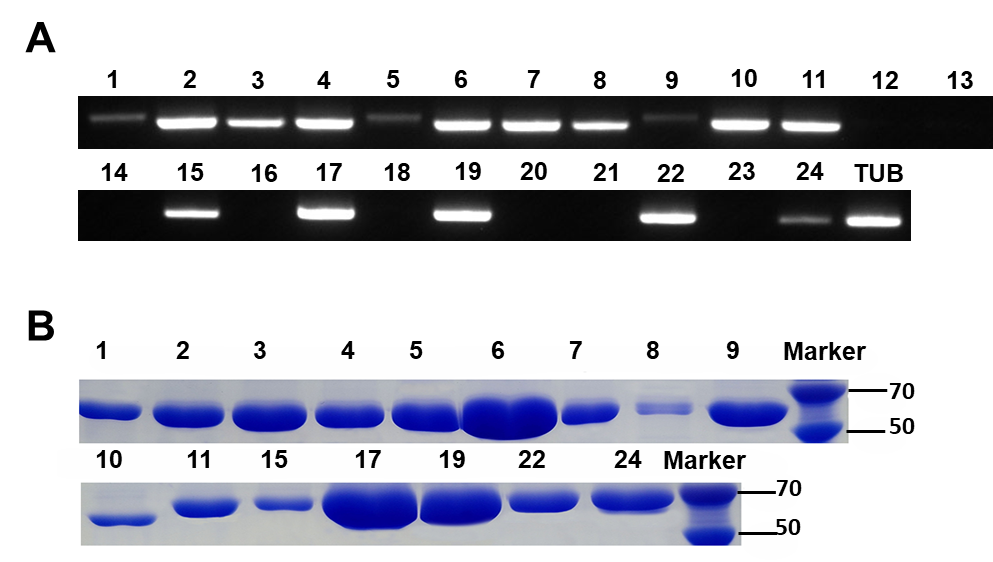
**

**Fig. S1. Identification of the PbrRALFs in pear pollen.**

A. RT-PCR expression analysis of PbrRALF1–PbrRALF24 (1–24) in pear pollen. TUB is used as a reference gene.

B. Recombinant proteins for the PbrRALFs corresponding to the 16 genes expressed in pear pollen were expressed and purified using the *E. coli* system.


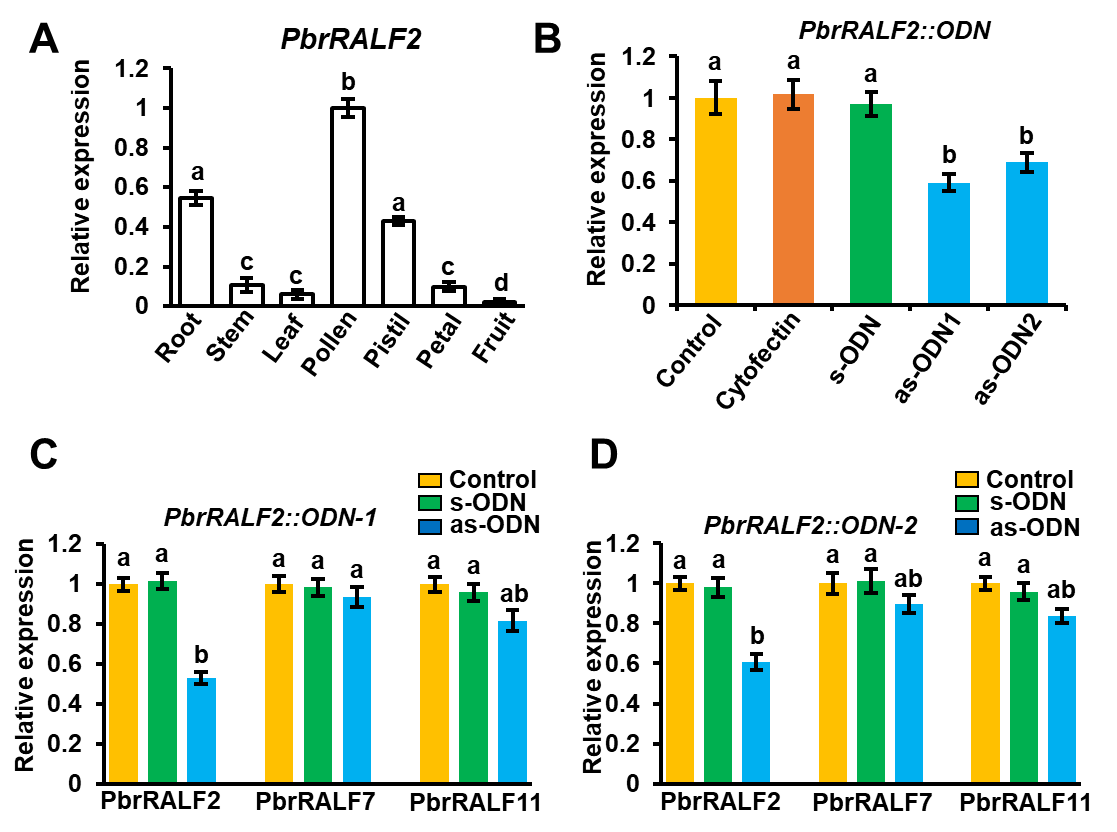


**Fig. S2. PbrRALF2-ODN treatments specifically knocked down the expression of PbrRALF2**

A. RT–qPCR analysis of the expression of PbrRALF2 in the seven indicated tissues of pear. PbrTUB is used as the reference gene.

B. Expression of PbrRALF2 was decreased after the as-ODN treatments. Cytofection and s-ODN were used as negative controls. UT indicates no treatment controls.

C. Expression levels of PbrRALF7 and PbrRALF11 were not affected by the PbrRALF2-ODN-1 treatment.

D. Expression levels of PbrRALF7 and PbrRALF11 were not affected by the PbrRALF2-ODN-2 treatment.


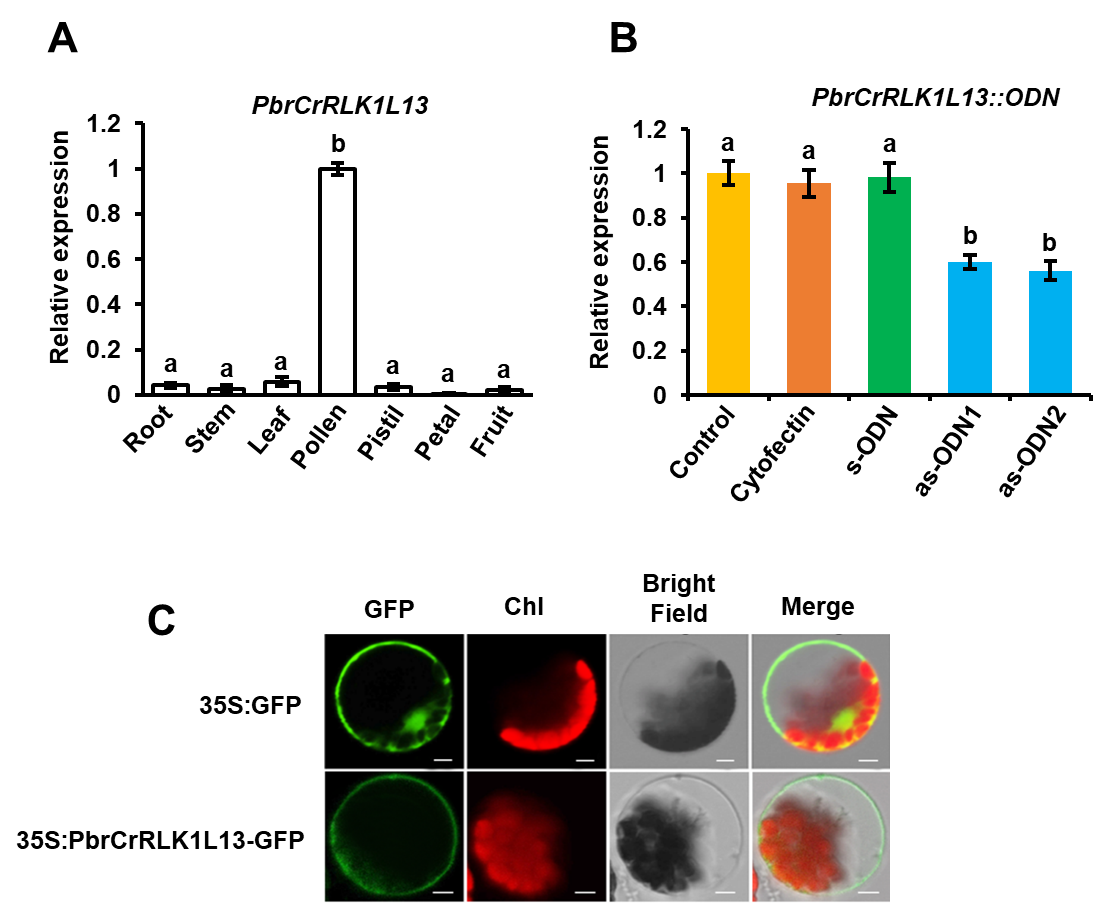


**Fig. S3. Expression and subcellular localization of PbrCrRLK1L13**

A. RT-qPCR analysis of the expression of PbrCrRLK1L13 in the seven indicated tissues of pear. PbrTUB is used as the reference gene.

B. Expression level of PbrCrRLK1L13 was decreased after the as-ODN treatment. Cytofection and s-ODN are used as controls.

C. PbrCrRLK1L13 is localized to the plasma membrane in Arabidopsis protoplasts. Bar = 10 μm.

**
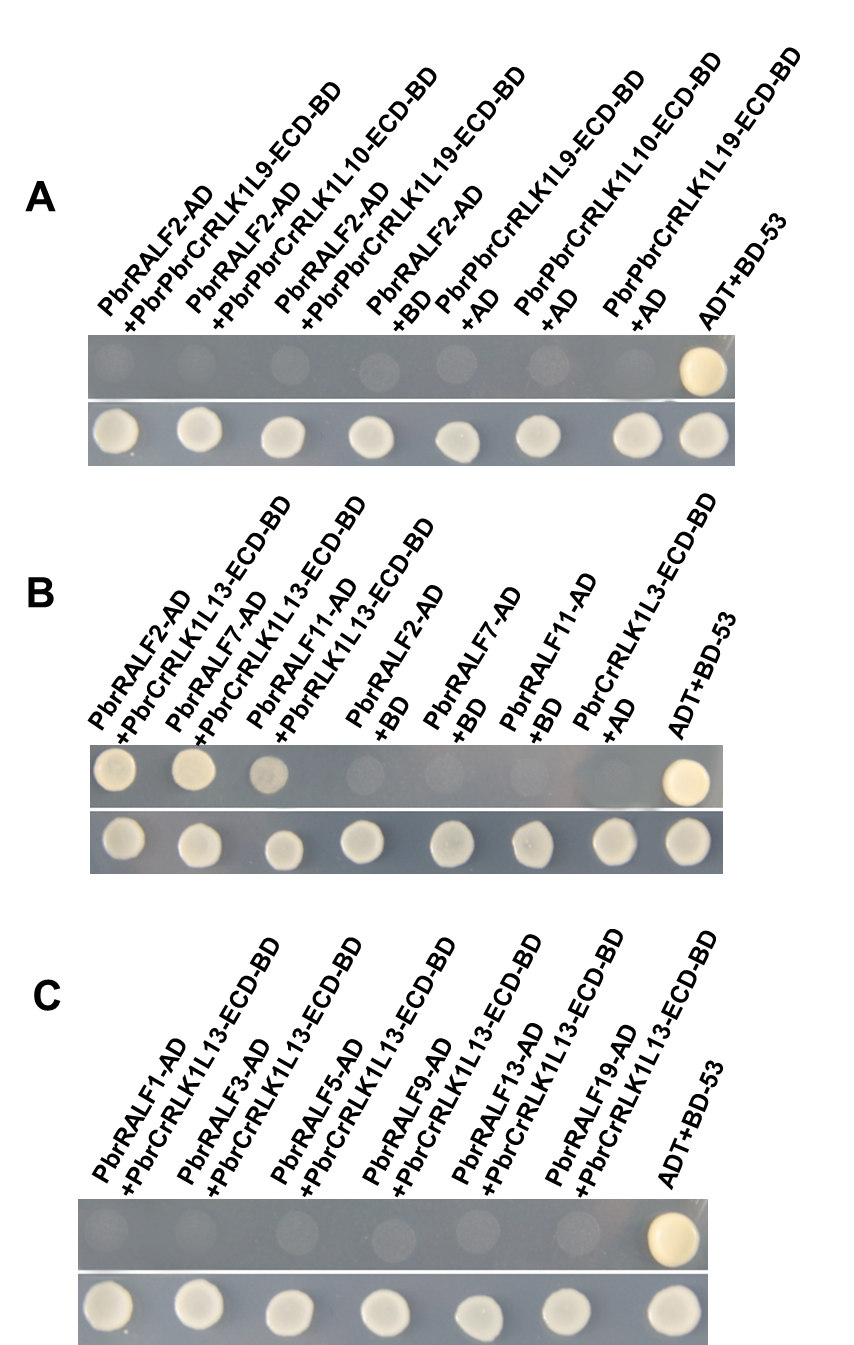
**

**Fig. S4. Yeast two-hybrid experiment testing the specificity of the interaction between PbrRALF2 and PbrCrRLK1L13.**

A. PbrRALF2 does not interact with three selected PbrCrRLK1Ls, including PbrCrRLK1L19 (homolog of THE1 in *Arabidopsis*), PbrCrRLK1L10 (homolog of HERK1 in *Arabidopsis*) and PbrCrRLK1L9 (homolog of HERK2 in *Arabidopsis*).

B. PbrCrRLK1L13 could interact with PbrRALF2, PbrRALF7, and PbrRALF11.

C. PbrCrRLK1L13 does not interact with six other randomly selected PbrRALFs that have no significant inhibitory function on pollen tube growth.


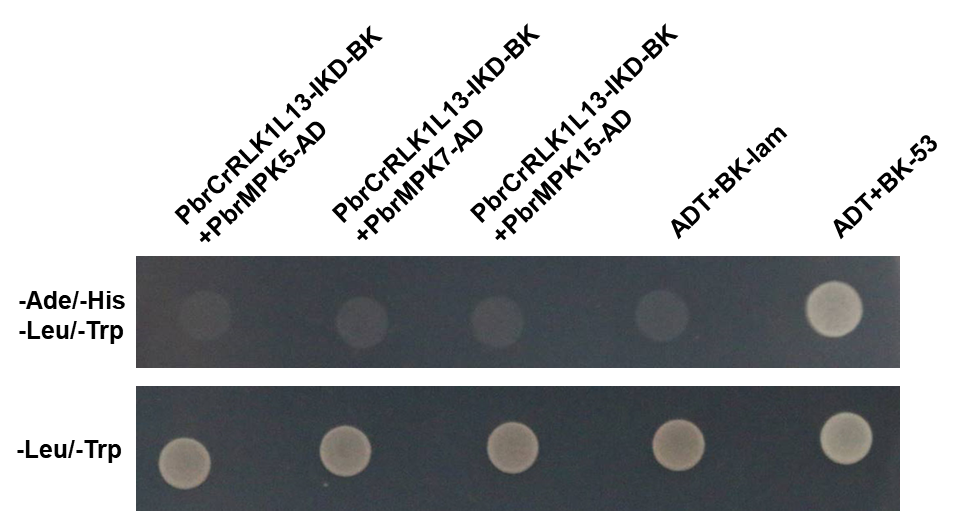


**Fig. S5. Yeast two-hybrid experiment testing the specificity of the interaction between PbrCrRLK1L13 and PbrMPK18**

Yeast two-hybrid experiments showed no interaction between PbrCrRLK1L13 and three other randomly selected PbrMPKs, including PbrMPK5, PbrMPK7, and PbrMPK15.


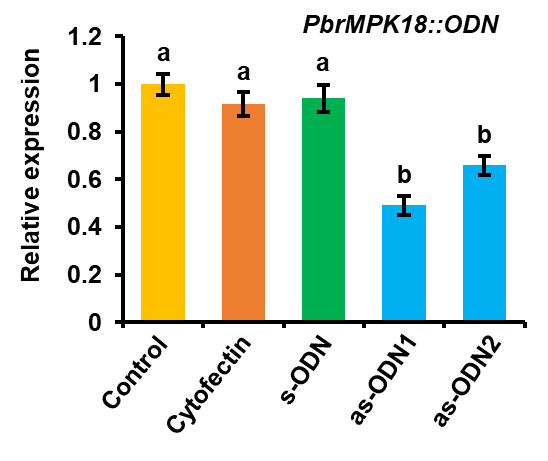


**Fig. S6. Expression level of PbrMPK18 was decreased after as-ODN treatment.**

Cytofection and s-ODN were used as negative controls. UT indicates no treatment control.

**
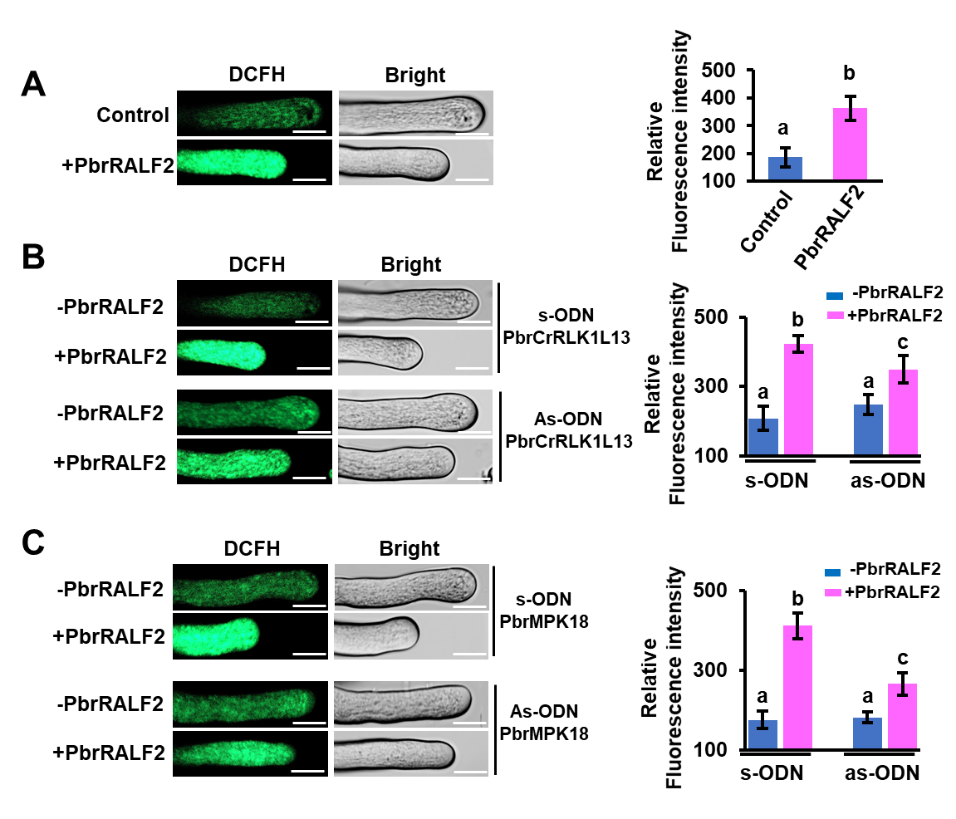
**

**Fig. S7. Reactive oxygen species (ROS) production induced by PbrRALF2 was detected using 2′,7′-dichlorodihydrofluorescein (CM-H2DCFDA).**

A. PbrRALF2 induces ROS production in pollen tubes. Representative images of pollen tubes incubated with 2′,7′-dichlorodihydrofluorescein (CM-H2DCFDA) under different treatments. Experiments were repeated at least three times with similar results. Bar = 50 μm. Different letters indicate significant differences, as determined by one-way ANOVA.

B. PbrRALF2 did not increase ROS in the as-ODN-treated PbrCrRLK1L13 pollen tube tips. Thirty-five pollen tubes were measured in each of three independent experiments. Bar = 50 μm. Different letters indicate significant differences, as determined by two-ANOVA.

C. PbrRALF2 did not increase ROS in the as-ODN-treated PbrMPK18 pollen tube tips. Thirty-five pollen tubes were measured in each of three independent experiments. Bar = 50 μm. Different letters indicate significant differences, as determined by two-way ANOVA.

| **Table S1. Summary of the structure of the RALF family in pear** | | | | | |
| --- | --- | --- | --- | --- | --- |
| Gene Name | Gene ID | Size (precursor protein) | CDS Size(bp) | Chr | pI |
| PbRALF1 | Pbr025155.1 | 134 | 405 | 2 | 6.11 |
| PbRALF2 | Pbr020387.1 | 120 | 363 | 4 | 8.52 |
| PbRALF3 | Pbr016493.1 | 112 | 339 | 4 | 8.97 |
| PbRALF4 | Pbr016486.1 | 115 | 358 | 4 | 8.18 |
| PbRALF5 | Pbr003781.1 | 62 | 189 | 5 | 9.2 |
| PbRALF6 | Pbr000376.1 | 123 | 372 | 5 | 9.96 |
| PbRALF7 | Pbr029069.1 | 72 | 219 | 9 | 9.13 |
| PbRALF8 | Pbr005197.1 | 214 | 645 | 9 | 9.42 |
| PbRALF9 | Pbr016245.1 | 121 | 366 | 10 | 9.35 |
| PbRALF10 | Pbr014940.1 | 69 | 210 | 10 | 9.26 |
| PbRALF11 | Pbr014856.1 | 126 | 381 | 13 | 9.75 |
| PbRALF12 | Pbr035438.1 | 134 | 405 | 15 | 6.72 |
| PbRALF13 | Pbr040194.1 | 134 | 405 | 15 | 7.6 |
| PbRALF14 | Pbr012076.1 | 115 | 348 | 16 | 5.73 |
| PbRALF15 | Pbr012077.1 | 69 | 213 | 16 | 8.49 |
| PbRALF16 | Pbr012079.1 | 115 | 348 | 16 | 5.78 |
| PbRALF17 | Pbr026835.1 | 109 | 330 | 17 | 4.5 |
| PbRALF18 | Pbr009637.1 | 112 | 339 | scaffold160.2 | 8.16 |
| PbRALF19 | Pbr034082.1 | 113 | 342 | scaffold633.0 | 7.62 |
| PbRALF20 | Pbr035711.1 | 134 | 405 | scaffold697.0 | 6.11 |
| PbRALF21 | Pbr004117.1 | 109 | 330 | scaffold1198.0 | 4.5 |
| PbRALF22 | Pbr004503.1 | 115 | 348 | scaffold1209.0 | 5.75 |
| PbRALF23 | Pbr004504.1 | 70 | 216 | scaffold1209.0 | 7.65 |
| PbRALF24 | Pbr004506.1 | 115 | 348 | scaffold1209.0 | 6.53 |

| **Table S2. Predicted protein selected by yeast two-hybrid using PbrRALF2 as a bait** | |
| --- | --- |
| Gene ID | Predicted protein |
| Pbr019497.1 | PROTEIN S-ACYL TRANSFERASE |
| Pbr015746.1 | E3 ubiquitin protein ligase RIN2 |
| Pbr040616.1 | Protein EXORDIUM-like 2 |
| Pbr001839.1 | CrRLK1L protein (PbrCrRLK1L13) |
| Pbr026118.1 | Defensin-like protein |
| Pbr028983.1 | Defensin-like protein |
| Pbr039965.1 | WRKY transcription factor 23 |
| Pbr035830.1 | Omega-6 fatty acid desaturase (FAD) |
| Pbr039965.1 | LRR receptor-like serine/threonine-protein kinase |

| **Table S3. Predicted protein selected by yeast two-hybrid using the IKD**  **of PbrCrRLK1L13 as a bait** | | |
| --- | --- | --- |
| Gene ID | Predicted protein |  |
| Pbr032029.1 | Protein phosphatase 2C-like |  |
| Pbr034199.1 | MAP kinase |  |
| Pbr007392.1 | MAP kinase (PbrMPK18) |  |
| Pbr037398.1 | Auxin responsive SAUR protein |  |
| Pbr028810.1 | Mannose-6-phosphate isomerase |  |
| Pbr037426.1 | Aldehyde dehydrogenase |  |
| Pbr019246.1 | Omega-6 fatty acid desaturase |  |
| Pbr021809.1 | Glycoside hydrolase |  |
| Pbr022368.1 | Arginine decarboxylase |  |
| Pbr020445.2 | ATMPK17 |  |
| Pbr042709.1 | Glucosamine/galactosamine-6-phosphate isomerase |  |
| Pbr013912.1 | Alcohol dehydrogenase |  |
| Pbr024288.1 | Cysteine desulfurase |  |

| **Table S4. Primers for His-PbrRALFs protein prokaryotic expression** | | |
| --- | --- | --- |
| Name | F (red indicates BamHI sites) | R (red indicates XbaI sites) |
| PbrRALF1-pCold | CGGGATCCATGGCGCGCAGATCTCTGTTCT | GCTCTAGATCGTCTGCATCGGGTG |
| PbrRALF2-pCold | CGGGATCCATGGAGTTTGACATGGACTCG | GCTCTAGAACTCCTGCACCTTGTGATG |
| PbrRALF3-pCold | CGGGATCCATGCTGCTGATGGAGTCGGAGAT | GCTCTAGACGAGTCCGACCTACAACG |
| PbrRALF4-pCold | CGGGATCCATGCCGGAGATGGAGTCAGAGAGC | GCTCTAGAATTACCTCTTGCACATCCCG |
| PbrRALF5-pCold | CGGGATCCATGGTGCTAAACTCCGAAGTCAAAG | GCTCTAGAATCCTGACCATGGCGAC |
| PbrRALF6-pCold | CGGGATCCATGCATCGGCCAAGGTCCAC | GCTCTAGAGCGTCTGGCGCAGC |
| PbrRALF7-pCold | CGGGATCCATGTGCTCAGTTTGCATCGAAACAG | GCTCTAGATTTCAATTTACGAACACCTCGG |
| PbrRALF8-pCold | CGGGATCCATGATCTCTGCAATTTTTGTTGAAGTAG | GCTCTAGAGCGGCATCGTTGTTCA |
| PbrRALF9-pCold | CGGGATCCATGGAGTTGTTGATGGATTCAAACAG | GCTCTAGAGCGTCTGGAGCAGCG |
| PbrRALF10-pCold | CGGGATCCATGATTACCTTGGTGGTGCTAAACTC | GCTCTAGAATTACCATCGCGACACC |
| PbrRALF11-pCold | CGGGATCCATGGAGATGCTGATGGACTCGGAG | GCTCTAGAGCGCCTGCAGCGAGT |
| PbrRALF15-pCold | CGGGATCCATGGAGTTTGGCATGGACTCGG | GCTCTAGAACTCCGGCAACGAGC |
| PbrRALF17-pCold | CGGGATCCATGCGTTGCGATGGC | GCTCTAGATCCGTCCCTTGCAC |
| PbrRALF19-pCold | CGGGATCCATGGGCATGGACTCAGAGATCAGC | GCTCTAGAACCCCGGCAACGG |
| PbrRALF22-pCold | CGGGATCCATGGAGTTTGGCATGGACTCGGAG | GCTCTAGAACTTCGGCAACGAGCATC |
| PbrRALF24-pCold | CGGGATCCATGGAGTTTGGCATGGACTCGG | GCTCTAGAACTCCGGCAACGAGTATT |
| PbrCrRLK1L13-ECD-pClod | CGGGATCCATGGCGTCCTCCCCACC | GCTCTAGACTTGTGAGGCCCGCCG |

| **Table S5. Primers for RT-PCR** | | |
| --- | --- | --- |
| Gene | Forward | Reverse |
| PbrRALF1 | CACTCTCCTCCTCTGCTGCT | GTCCGGTTCCAAGTCCTCG |
| PbrRALF2 | GCACCACCTCACCTGGATAC | GGAGCAAGGCACGGTGTTC |
| PbrRALF3 | AGTGTTCCTTCTGCTAATCATCG | GCCCCAGGCGATATGTACTTC |
| PbrRALF4 | TCAAAGGGAGCGGAATCGAG | TTCCACATGGAACCGAGTC |
| PbrRALF5 | ATGGTGGTGCTAAACTCCGA | TAATCCTGACCATGGCGACAC |
| PbrRALF6 | TCGGCGATCACGATGATGAG | TGCCGCGGCCTCTGA |
| PbrRALF7 | TCCAAGACGTTTACACTTTGC | TTACGAACACCTCGGCACTT |
| PbrRALF8 | CCTTGGCACTCTCCTCATCTC | GCATAATCCGACCATTGCGGC |
| PbrRALF9 | GTGGCTCATGATGATGAATCCTTG | GTTGTAGTAGGACTGGCCGC |
| PbrRALF10 | GTCATTCATCCTTTGTTTCCTCCT | TCGCGACACCAAAGCATATT |
| PbrRALF11 | CGATTGCTTCGGCCGTTGA | GTGTTCCTCCTCAGCGCAC |
| PbrRALF12 | GCAACCACTCTTTTCCTCTGC | CGTCCAGTTCCAAGTCCTCG |
| PbrRALF13 | CCGACCGCCCCAATGTC | GGATGCGATTCGCCGACAG |
| PbrRALF14 | CTGCGAGGGTTCTGTAGCA | GAGCGCAAGGCACAGAGTT |
| PbrRALF15 | CACCTCGCGAAACATAAGCTA | GGCAACGAGCATTAGCACTG |
| PbrRALF16 | GAGCTGGGTTCCAGTGAGAC | CTTATGTTTCGCGAGGTGGC |
| PbrRALF17 | ACCACTGGATCGTTGCGATG | ATCCCTCTGTAGGGCTCCAT |
| PbrRALF18 | GTCTTCCCTCTGATAGTCCTCG | GCCCCAGGTGATATGTACTTC |
| PbrRALF19 | TTGGCCACCTCACAGTACAT | GGGGTTGCTCTGGGCAC |
| PbrRALF20 | TCACTCTCCTCCTCTGCTGC | AGACCCTCCGTCACTAGCTT |
| PbrRALF21 | GGCGTAATGCCGGTGAAGTA | CTCCTCGTTGCCCTCCTCT |
| PbrRALF22 | ACTCAAAGGGAGCGGAATCG | ATCCTCCTGCTGCTCTCTGA |
| PbrRALF23 | GGACTCGGAGATTAGCAGGC | TGGAGGAAGCACCTCTCTGA |
| PbrRALF24 | TCATGGGGTGAGCTGTGTTC | TTCGTGAGGTGGCCAAGATG |
| PbrCrRLK1L13 | TCCGTGGACAGTTTGGATGG | ACCATTGCTCCAAGCCCTAC |
| PbrTUB | TGGGCTTTGCTCCTCTTAC | CCTTCGTGCTCATCTTACC |

| **Table S6. Primers for qRT-PCR** | | | |
| --- | --- | --- | --- |
| Gene | | Forward | Reverse |
| PbrRALF2 | GCACCACCTCACCTGGATAC | | GGAGCAAGGCACGGTGTTC |
| PbrRALF7 | TCCAAGACGTTTACACTTTGC | | TTACGAACACCTCGGCACTT |
| PbrRALF11 | CGATTGCTTCGGCCGTTGA | | GTGTTCCTCCTCAGCGCAC |
| PbrCrRLK1L13 | TCCGTGGACAGTTTGGATGG | | ACCATTGCTCCAAGCCCTAC |
| PbrMPK18 | CCGCCACATCAGGCGAT | | AACGTGCTCGTTCGTCTCC |
| PbrTUB | TGGGCTTTGCTCCTCTTAC | | CCTTCGTGCTCATCTTACC |

| **Table S7. Primers for yeast two-hybrid** | |
| --- | --- |
| Name | Primer (red indicates restriction enzyme sites) |
| PbrRALF2-AD-F-EcoRI | CGGAATTCATGGAGTTTGACATGGACTCG |
| PbrRALF2-AD-R-BamHI | CGGGATCCACTCCTGCACCTTGTGATG |
| PbrRALF7-AD-F- EcoRI | CGGAATTCATGTGCTCAGTTTGCATCGAAACAG |
| PbrRALF7-AD-R-BamHI | CGGGATCCTTTCAATTTACGAACACCTCGGC |
| PbrRALF11-AD-F- EcoRI | CGGAATTCATGGAGATGCTGATGGACTCGGAG |
| PbrRALF11-AD-R-BamHI | CGGGATCCGCGCCTGCAGCGAGTAAT |
| PbrRALF19-AD-F- EcoRI | CGGAATTCATGGGCATGGACTCAGAGATCAGC |
| PbrRALF19-AD-R-BamHI | CGGGATCCACCCCGGCAACGGGC |
| PbrRALF5-AD-F- EcoRI | CGGAATTCATGGTGCTAAACTCCGAAGTCAAAGG |
| PbrRALF5-AD-R-BamHI | CGGGATCCATCCTGACCATGGCGACACC |
| PbrRALF9-AD-F- EcoRI | CGGAATTCATGGAGTTGTTGATGGATTCAAACAGC |
| PbrRALF9-AD-R-BamHI | CGGGATCCGCGTCTGGAGCAGCGGG |
| PbrCrRLK1L13-ECD-BK-F-BamHI | CGGGATCCATGGCGTCCTCCCCACC |
| PbrCrRLK1L13-ECD-BK-R-PstI | AACTGCAGCTTGTGAGGCCCGCCG |
| PbrCrRLK1L19-ECD-BK-F-EcoRI | CGGAATTCATGTCATTCACGGCTGTCG |
| PbrCrRLK1L19-ECD-BK-R-BamHI | CGGGATCCTCCTATATTATTCTTCTTTGAGGAT |
| PbrCrRLK1L10-ECD-BK-F-EcoRI | CGGAATTCATGGACAATTACCTTATAGACTGTGGAT |
| PbrCrRLK1L10-ECD-BK-R-SalI | ACGCGTCGACAACGTTACTTTTTGAACCTGGATTC |
| PbrCrRLK1L9-ECD-BK-F-BamHI | CGGGATCCATGGAAGCTCAATCAAAATCTTTTC |
| PbrCrRLK1L9-ECD-BK-R-SalI | ACGCGTCGACTTGATTTTTTGAACTCCGTGGTG |
| PbrCrRLK1L13(31–187)-F-BamHI | CGGGATCCATGAACTTCCTCATCGATTGTG |
| PbrCrRLK1L13(31–187)-R-PstI | AACTGCAGATCAGGAGCTGAAACCACCT |
| PbrCrRLK1L13(31–212)-F-BamHI | CGGGATCCATGAACTTCCTCATCGATTGTG |
| PbrCrRLK1L13(31–212)-R-PstI | AACTGCAGTTCGTACCCAAATTTGGACAG |
| PbrCrRLK1L13(31–390)-F-BamHI | CGGGATCCATGAACTTCCTCATCGATTGTG |
| PbrCrRLK1L13(31–390)-R-PstI | AACTGCAGTAGTTTCATCACCTCCAAACCA |
| PbrCrRLK1L13(31–417)-F-BamHI | CGGGATCCATGAACTTCCTCATCGATTGTGG |
| PbrCrRLK1L13(31–417)-R-PstI | AACTGCAGCTTGTGAGGCCCGCCG |
| PbrCrRLK1L13(213–390)-F-BamHI | CGGGATCCATGACAATGTACAGGCTTAACATGG |
| PbrCrRLK1L13(213–390)-R-PstI | AACTGCAGTAGTTTCATCACCTCCAAACCAT |
| PbrCrRLK1L13(391–417)-F-BamHI | CGGGATCCATGAGCAATTCCGTGGACAGTTT |
| PbrCrRLK1L13(391–417)-R-PstI | AACTGCAGCTTGTGAGGCCCGCCG |
| PbrCrRLK1L13-IKD-AD-F-EcoRI | CGGAATTCATGAAATGGCGAAAGAGACCT |
| PbrCrRLK1L13-IKD-AD-R-XhoI | CCGCTCGAGCCTACCATTTAGATTGGAAAATTG |
| PbrMPK18-BK-F-SmaI | TCCCCCGGGATGGAGGGAGGAGGGCGAT |
| PbrMPK18-BK-R-BglII | GAAGATCTCAGTCGCTGATACTCGGGGTT |

| **Table S8. Primers for BIFC** | |
| --- | --- |
| Name | Primer (red indicates restriction enzyme sites) |
| PbrCrRLK1L13-BiFC-F-XbaI | GCTCTAGAATGGCTCTCCTCCTGGTCC |
| PbrCrRLK1L13-BiFC-R-BglII | GAAGATCTCCTACCATTTAGATTGGAAAATTG |
| PbrMPK18-BiFC-F-XbaI | GCTCTAGAATGGAGGGAGGAGGGCGAT |
| PbrMPK18-BiFC-R-BglII | GAAGATCTCAGTCGCTGATACTCGGGGTT |

| **Table S9. Primers for LCI** | |
| --- | --- |
| Name | Primer (red indicates homology arm (containing restriction enzyme sites) |
| PbrCrRLK1L13-nLUC-F-BamHI | CTCGGTACCCGGGGATCCATGGCTCTCCTCCTGGTCC |
| PbrCrRLK1L13-nLUC-R-SalI | GTACGAGATCTGGTCGACCCTACCATTTAGATTGGAAAATTG |
| PbrMPK18-cLUC-F-KpnI | GCGTCCCGGGGCGGTACCATGGAGGGAGGAGGGCGAT |
| PbrMPK18-cLUC-R-BamHI | AGTCCATTTGTTGGATCCCAGTCGCTGATACTCGGGGTT |

| **Table S10. Sequences for ODN** | | |
| --- | --- | --- |
| Name | Antisense-ODN | Sense-ODN |
| PbrCrRLK1L13-ODN1 | GCAGCCACCGCACCCTT | ACTAGGTCTCGTGCGTACGC |
| PbrCrRLK1L13-ODN2 | GTACCCGAAGCTTCCCT |  |
| PbrRALF2-ODN1 | GTGATGGCGCTGCAGCCGCGG | GGAGATCAGCCGTCGCA |
| PbrRALF2-ODN2 | GCGCACCGTAGCTGATGT |  |
| PbrMPK18-ODN1 | GTGGCCGGAATGCTCTC | TACGCGGAAATGGCTACCCG |
| PbrMPK18-ODN2 | CGTCCTCTTCGCATCGATCC |  |

Note: red indicates phosphorothioate modification sites

**Data S1. ANOVA tables**

(Sum Sq, sum of squares; df, degrees of freedom; Mean Sq, mean squares)

| Fig. 1A | | One-way ANOVA | |  | |  | |  | |  | |  | |
| --- | --- | --- | --- | --- | --- | --- | --- | --- | --- | --- | --- | --- | --- |
|  | | ANOVA table | | Sum Sq | | df | | Mean Sq | | F-value | | *p* value | |
|  | | Treatment | | 384570 | | 16 | | 24035.6 | | 171.14 | | *p <*0.01 | |
|  | | Residual | | 4473.4 | | 32 | | 139.8 | |  | |  | |
|  | | Total | | 389043 | | 48 | |  | |  | |  | |
|  | |  | |  | |  | |  | |  | |  | |
| Fig. 1C | | One-way ANOVA | |  | |  | |  | |  | |  | |
|  | | ANOVA table | | Sum Sq | | df | | Mean Sq | | F-value | | *p* value | |
|  | | Treatment | | 88420.3 | | 14 | | 6315.7 | | 3.84 | | *p <*0.05 | |
|  | | Residual | | 2338.1 | | 18 | | 124.3 | |  | |  | |
|  | | Total | | 90758.4 | | 32 | |  | |  | |  | |
|  | |  | |  | |  | |  | |  | |  | |
| Fig. 2B | | One-way ANOVA | |  | |  | |  | |  | |  | |
|  | | ANOVA table | | Sum Sq | | df | | Mean Sq | | F-value | | *p* value | |
|  | | Treatment | | 456.3 | | 6 | | 76.1 | | 18.7 | | *p <*0.05 | |
|  | | Residual | | 28.6 | | 8 | | 3.57 | |  | |  | |
|  | | Total | | 484.9 | | 14 | |  | |  | |  | |
|  | |  | |  | |  | |  | |  | |  | |
| Fig. 3B | | One-way ANOVA | |  | |  | |  | |  | |  | |
|  | | ANOVA table | | Sum Sq | | df | | Mean Sq | | F-value | | *p* value | |
|  | | Treatment | | 65236.4 | | 18 | | 3624.2 | | 135.6 | | *p <*0.01 | |
|  | | Residual | | 961.8 | | 22 | | 43.7 | |  | |  | |
|  | | Total | | 66198.2 | | 40 | |  | |  | |  | |
|  | |  | |  | |  | |  | |  | |  | |
| Fig. 3D | | One-way ANOVA | |  | |  | |  | |  | |  | |
|  | | ANOVA table | | Sum Sq | | df | | Mean Sq | | F-value | | *p* value | |
|  | | Treatment | | 4683.1 | | 21 | | 223 | | 46.6 | | *p <*0.01 | |
|  | | Residual | | 200.7 | | 26 | | 7.7 | |  | |  | |
|  | | Total | | 4883.8 | | 47 | |  | |  | |  | |
|  | |  | |  | |  | |  | |  | |  | |
| Fig. 4A | | One-way ANOVA | |  | |  | |  | |  | |  | |
|  | | ANOVA table | | Sum Sq | | df | | Mean Sq | | F-value | | *p* value | |
|  | | Treatment | | 63948.8 | | 20 | | 3197.44 | | 73.78 | | *p <*0.05 | |
|  | | Residual | | 1733.5 | | 26 | | 66.7 | |  | |  | |
|  | | Total | | 65682.3 | | 46 | |  | |  | |  | |
|  | |  | |  | |  | |  | |  | |  | |
| Fig. 4B | | Two-way ANOVA | |  | |  | |  | |  | |  | |
|  | | ANOVA table | | Sum Sq | | df | | Mean Sq | | F-value | | *p* value | |
|  | | Interaction | | 2592.6 | | 8 | | 324.1 | | 76.3 | | *p <*0.01 | |
|  | | Row Factor | | 4498.5 | | 4 | | 1124.6 | | 264.7 | | *p <*0.01 | |
|  | | Column Factor | | 6355.7 | | 2 | | 3177.8 | | 187.1 | | *p <*0.01 | |
|  | | Residual | | 1699.1 | | 31 | | 54.8 | |  | |  | |
|  | |  | |  | |  | |  | |  | |  | |
| Fig. 4C | | One-way ANOVA | |  | |  | |  | |  | |  | |
|  | | ANOVA table | | Sum Sq | | df | | Mean Sq | | F-value | | *p* value | |
|  | | Treatment | | 26722 | | 12 | | 2226.8 | | 121.76 | | *p* *<*0.01 | |
|  | | Residual | | 438.9 | | 8 | | 54.86 | |  | |  | |
|  | | Total | | 27160.9 | | 20 | |  | |  | |  | |
|  | |  | |  | |  | |  | |  | |  | |
| Fig. 4D | | Two-way ANOVA | |  | |  | |  | |  | |  | |
|  | | ANOVA table | | Sum Sq | | df | | Mean Sq | | F-value | | *p* value | |
|  | | Interaction | | 5633.94 | | 4 | | 1408.45 | | 46.38 | | *p* *<*0.01 | |
|  | | Row Factor | | 7106.48 | | 2 | | 3553.24 | | 117 | | *p <*0.01 | |
|  | | Column Factor | | 6175.91 | | 2 | | 3088 | | 50.84 | | *p <*0.01 | |
|  | | Residual | | 607.37 | | 16 | | 37.96 | |  | |  | |
|  | |  | |  | |  | |  | |  | |  | |
| Fig. 6B | | Two-way ANOVA | |  | |  | |  | |  | |  | |
|  | | ANOVA table | | Sum Sq | | df | | Mean Sq | | F-value | | *p* value | |
|  | | Interaction | | 3546.24 | | 8 | | 443.28 | | 63.33 | | *p <*0.01 | |
|  | | Row Factor | | 5080.72 | | 3 | | 1693.57 | | 181.47 | | *p <*0.01 | |
|  | | Column Factor | | 3363.11 | | 3 | | 1121.03 | | 60.06 | | *p <*0.01 | |
|  | | Residual | | 279.98 | | 18 | | 15.55 | |  | |  | |
|  | |  | |  | |  | |  | |  | |  | |
| Fig. 6C | | Two-way ANOVA | |  | |  | |  | |  | |  | |
|  | | ANOVA table | | Sum Sq | | df | | Mean Sq | | F-value | | *p* value | |
|  | | Interaction | | 37475.4 | | 7 | | 5353.62 | | 157.77 | | *p <*0.01 | |
|  | | Row Factor | | 38376.2 | | 4 | | 9594.04 | | 323.12 | | *p <*0.01 | |
|  | | Column Factor | | 43590.9 | | 2 | | 21795.4 | | 183.52 | | *p <*0.01 | |
|  | | Residual | | 1187.66 | | 21 | | 56.55 | |  | |  | |
|  | |  | |  | |  | |  | |  | |  | |
| Fig. S2A | | One-way ANOVA | |  | |  | |  | |  | |  | |
|  | | ANOVA table | | Sum Sq | | df | | Mean Sq | | F-value | | *p* value | |
|  | | Treatment | | 138.29 | | 6 | | 23.05 | | 33.19 | | *p <*0.05 | |
|  | | Residual | | 0.84 | | 12 | | 0.07 | |  | |  | |
|  | | Total | | 139.13 | | 18 | |  | |  | |  | |
|  | |  | |  | |  | |  | |  | |  | |
| Fig. S2B | | One-way ANOVA | |  | |  | |  | |  | |  | |
|  | | ANOVA table | | Sum Sq | | df | | Mean Sq | | F-value | | *p* value | |
|  | | Treatment | | 0.75 | | 4 | | 0.19 | | 39.42 | | *p <*0.05 | |
|  | | Residual | | 0.04 | | 8 | | 0.005 | |  | |  | |
|  | | Total | | 0.79 | | 12 | |  | |  | |  | |
|  | |  | |  | |  | |  | |  | |  | |
| Fig. S2C | | Two-way ANOVA | |  | |  | |  | |  | |  | |
|  | | ANOVA table | | Sum Sq | | df | | Mean Sq | | F-value | | *p* value | |
|  | | Interaction | | 0.1647 | | 4 | | 0.041 | | 0.08 | | *p <*0.01 | |
|  | | Row Factor | | 0.09 | | 2 | | 0.045 | | 4.11 | | *p <*0.01 | |
|  | | Column Factor | | 1.281 | | 2 | | 0.64 | | 0.01 | | *p <*0.01 | |
|  | | Residual | | 0.17 | | 16 | | 0.011 | |  | |  | |
|  | |  | |  | |  | |  | |  | |  | |
| Fig. S2D | | Two-way ANOVA | |  | |  | |  | |  | |  | |
|  | | ANOVA table | | Sum Sq | | df | | Mean Sq | | F-value | | *p* value | |
|  | | Interaction | | 0.2864 | | 4 | | 0.0716 | | 0.68 | | *p <*0.01 | |
|  | | Row Factor | | 0.4513 | | 2 | | 0.225 | | 3.94 | | *p <*0.01 | |
|  | | Column Factor | | 2.523 | | 2 | | 1.261 | | 0.02 | | *p <*0.01 | |
|  | | Residual | | 0.2241 | | 14 | | 0.016 | |  | |  | |
|  | |  | |  | |  | |  | |  | |  | |
| Fig. S3A | | One-way ANOVA | |  | |  | |  | |  | |  | |
|  | | ANOVA table | | Sum Sq | | df | | Mean Sq | | F-value | | *p* value | |
|  | | Treatment | | 13.59 | | 6 | | 2.265 | | 291.6 | | *p <*0.05 | |
|  | | Residual | | 0.04 | | 12 | | 0.003 | |  | |  | |
|  | | Total | | 13.63 | | 18 | |  | |  | |  | |
|  | |  | |  | |  | |  | |  | |  | |
| Fig. S3B | | One-way ANOVA | |  | |  | |  | |  | |  | |
|  | | ANOVA table | | Sum Sq | | df | | Mean Sq | | F-value | | *p* value | |
|  | | Treatment | | 14.32 | | 4 | | 3.58 | | 48.56 | | *p <*0.05 | |
|  | | Residual | | 0.04 | | 8 | | 0.005 | |  | |  | |
|  | | Total | | 14.36 | | 12 | |  | |  | |  | |
|  | |  | |  | |  | |  | |  | |  | |
| Fig. S6 | | One-way ANOVA | |  | |  | |  | |  | |  | |
|  | | ANOVA table | | Sum Sq | | df | | Mean Sq | | F-value | | *p* value | |
|  | | Treatment | | 2.33 | | 4 | | 0.5825 | | 120.2 | | *p <*0.05 | |
|  | | Residual | | 0.04 | | 8 | | 0.005 | |  | |  | |
|  | | Total | | 2.37 | | 12 | |  | |  | |  | |
| Fig. S7A | | One-way ANOVA | |  | |  | |  | |  | |  |  |
|  | | ANOVA table | | Sum Sq | | df | | Mean Sq | | F-value | | *p* value |  |
|  | | Treatment | | 6715.3 | | 14 | | 479.7 | | 73.1 | | *p <*0.01 |  |
|  | | Residual | | 361.2 | | 28 | | 12.9 | |  | |  |  |
|  | | Total | | 7076.5 | | 42 | |  | |  | |  |  |
| Fig. S7B | | Two-way ANOVA | |  | |  | |  | |  | |  |  |
|  | | ANOVA table | | Sum Sq | | df | | Mean Sq | | F-value | | *p* value |  |
|  | | Interaction | | 4325.9 | | 2 | | 2163.0 | | 56.3 | | *p* <0.01 |  |
|  | | Row Factor | | 3448.1 | | 2 | | 1724.1 | | 59.7 | | *p* <0.01 |  |
|  | | Column Factor | | 7343.7 | | 1 | | 7343.7 | | 119.1 | | *p* <0.01 |  |
|  | | Residual | | 1398.1 | | 12 | | 116.5 | |  | |  |  |
| Fig. S7C | | Two-way ANOVA | |  | |  | |  | |  | |  |  |
|  | | ANOVA table | | Sum Sq | | df | | Mean Sq | | F-value | | *p* value |  |
|  | | Interaction | | 10674.9 | | 4 | | 2668.7 | | 41.5 | | *p* <0.01 |  |
|  | | Row Factor | | 7546.5 | | 2 | | 3773.2 | | 86.4 | | *p* <0.01 |  |
|  | | Column Factor | | 6324.9 | | 2 | | 3162.5 | | 105.8 | | *p* <0.01 |  |
|  | | Residual | | 437.8 | | 16 | | 27.4 | |  | |  |  |
